# Dietary targeting of TRPM8 rewires macrophage immunometabolism reducing colitis severity

**DOI:** 10.1038/s41419-025-07553-9

**Published:** 2025-04-25

**Authors:** D. Cicia, F. Biscu, FA. Iannotti, M. Miraglia, C. Ferrante, N. Iaccarino, S. Cadenas de Miguel, A. Chiavaroli, A. Schiano Moriello, P. De Cicco, MF. Nanì, L. Zanoletti, B-J. Ke, L. van Baarle, K. Talavera, A. Randazzo, I. Elia, R. Capasso, G. Matteoli, E. Pagano, AA. Izzo

**Affiliations:** 1 Department of Pharmacy, School of Medicine and Surgery, University of Naples Federico II, Naples, Italy; 2 Laboratory of Mucosal Immunology, Translational Research Center for Gastrointestinal Disorders (TARGID), KU Leuven Leuven, Belgium; 3 Centre for Inflammation Research, University of Edinburgh, Edinburgh, UK; 4 Institute of Biomolecular Chemistry ICB, CNR, Pozzuoli, Naples, Italy; 5 Department of Pharmacy, Gabriele d’Annunzio University, Chieti, Italy; 6 Laboratory of Cellular Metabolism and Metabolic Regulation, Department of Oncology, KU Leuven and Leuven Cancer Institute, Leuven, Belgium; 7 Department of Biology and Biotechnology “L. Spallanzani”, University of Pavia, Pavia, Italy; 8 Laboratory of Ion Channel Research, Department of Cellular and Molecular Medicine, KU Leuven and VIB Center for Brain and Disease Research, Leuven, Belgium; 9 Department of Agricultural Sciences, University of Naples Federico II, Naples, Italy

**Keywords:** Mucosal immunology, Monocytes and macrophages, Chronic inflammation

## Abstract

The interplay between diet, host genetics, microbiota, and immune system has a key role in the pathogenesis of inflammatory bowel disease (IBD). Although the causal pathophysiological mechanisms remain unknown, numerous dietary nutrients have been shown to regulate gut mucosal immune function, being effective in influencing innate or adaptive immunity. Here, we proved that transient receptor potential melastatin 8 (TRPM8), a non-selective cation channel, mediates LPS- evoked Ca^2+^ influx in macrophages leading to their activation. Additionally, we showed that TRPM8 is selectively blocked by the dietary flavonoid luteolin, which induced a pro-tolerogenic phenotype in pro-inflammatory macrophages. Accordingly, genetic deletion of *Trpm8* in macrophages caused a deficit in the activation of pro-inflammatory metabolic and transcriptional reprogramming, leading to reduced production of key pro-inflammatory cytokines such as interleukin (IL)-1β, IL-6, and tumor necrosis factor (TNF)-α. The TRPM8 anti-inflammatory effect was found to be dependent on lactate which in turn induces IL-10 gene expression. Adoptive transfer of TRPM8-deficient bone marrow in wild-type mice improved intestinal inflammation in a model of colitis. Accordingly, oral administration of luteolin protected mice against colitis through an impairment in the innate immune response. Our study reveals the potential of targeting TRPM8 through specific nutrient interventions to regulate immune function in sub-clinical scenarios or to treat inflammatory diseases, primarily driven by chronic immune responses, such as IBD.

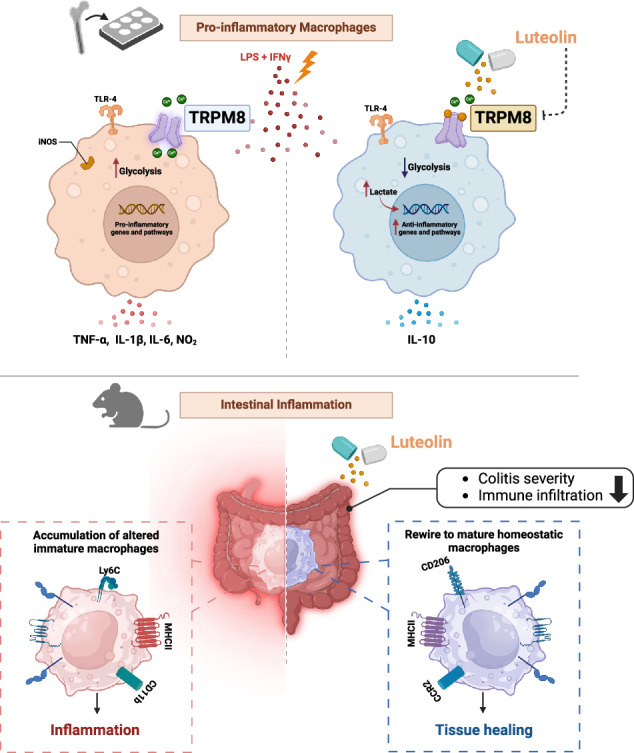

## Introduction

Numerous environmental factors (i.e., diet, smoking, medications) alter gut homeostasis, changing microbial composition, mucosal barrier function, and immune tolerance [1]. Dysregulation of gut immune function causes a defect in the resolution of inflammation, laying the basis for the development and progression of gut chronic inflammatory conditions, such as inflammatory bowel diseases (IBDs) [2, 3]. Diet may play a causative role in IBD, and as a matter of fact, dietary factors have been recently related to a dysregulated activation of the mucosal immune system in patients with IBD [1, 4]. Dietary manipulation [i.e., Crohn’s disease exclusion diet (CDED), Exclusive Enteral Nutrition (EEN), and Partial Enteral Nutrition (PEN)] is becoming essential in the management of IBD at different stages of pathology [5–9]. Furthermore, the International Organization for the Study of Inflammatory Bowel Disease (IOIBD) and the European Society for Clinical Nutrition and Metabolism (ESPEN) guidelines indicate that a “Mediterranean-like” diet improves IBD symptoms [10, 11]. Based on such findings, the scientific interest in diet and dietary supplementation has exponentially increased in the past ten years. In parallel, the search for dietary components that may further enhance immune function in sub-clinical situations or prevent specific immune-related chronic diseases may lead to new therapeutic strategies [12].

Consistent with epidemiologic observations, studies in IBD animal models confirm that specific factors in the diet may directly affect mucosal integrity and immune function [3, 12]. It has been shown that this can be achieved by triggering signaling pathways regulating the differentiation of macrophages [13], which, together with neutrophils, act as main regulators of inflammatory response. In response to tissue damage, macrophages lose immune tolerance promoting leukocyte recruitment, primarily neutrophils and monocytes [13, 14]. In the later stage of inflammation, macrophages acquire a pro-tolerogenic phenotype, preventing excessive immune response and promoting resolution of inflammation through the release of pro-resolutive cytokines, mainly interleukin (IL)-10. In IBD patients, the balance between effector and regulatory immune mechanisms is disturbed [15]. Pro-inflammatory monocyte-like cells accumulate in the inflamed gut of IBD patients, secreting high levels of pro-inflammatory cytokines [e.g., tumor necrosis factor (TNF)-α, IL-6, IL-23, and IL-1β], increasing the susceptibility to commensal microbiota and promoting the expansion of pro-inflammatory T-cells [16]. Given these insights, macrophage-targeting therapies emerge as a promising approach to recalibrate the intestinal immune milieu and reestablish tissue equilibrium following inflammation.

Within the possible molecular targets of dietary therapeutic interventions, Transient Receptor Potential (TRP) channels have significant potential, as they are directly modulated by a plethora of spices known to improve gastrointestinal functions[17]. One of the most relevant examples is Transient Receptor Potential Melastatin 8 (TRPM8), which is activated by menthol (from *Mentha x piperita*) and eucalyptol (from *Eucalyptus spp*.) [18, 19], two compounds having anti-inflammatory effects in many pathological conditions [20–22]. TRPM8 regulates the innate immune responses during intestinal inflammation [23–25], and it is up-regulated in the inflamed colon of mice and patients with Crohn’s disease (CD) [23, 24] as well as in activated murine peritoneal resident macrophages [25]. Furthermore, recent findings document an involvement of TRPM8 in human monocyte differentiation [26].

These precedents led us to hypothesize that targeting TRPM8 by dietary ligands may affect macrophage functional plasticity, being beneficial in the management of inflammatory diseases primarily mediated by innate immune responses. While testing this hypothesis, we unveiled a pivotal immunomodulatory role of TRPM8 and identified the dietary flavonoid luteolin as a new selective TRPM8 antagonist. Luteolin-induced inhibition of TRPM8 modulates the response of bone marrow-derived macrophages (BMDMs) to PAMPs (i.e., LPS and IFN-γ), diminishing their pro-inflammatory capacity and metabolic switch. This effect is downstream mediated by a lactate-dependent regulation of IL-10 activity. Accordingly, we demonstrated that genetic ablation of TRPM8 induces a pro-tolerogenic profile in macrophages in vitro, and reduces colitis susceptibility in vivo, revealing the critical importance of this receptor in innate immunity. Similarly, oral supplementation of luteolin ameliorates colitis in mice, through an impairment in the innate immune response, showing the possibility of novel pharmacological strategies for the treatment of chronic inflammatory gut disorders.

## Results

### Luteolin is a new selective TRPM8 blocker

Given the established association between TRPM8 activation and modulation of inflammatory responses [27], this study embarked on identifying novel dietary ligands for TRPM8 with potential anti-inflammatory properties.

**Fig. 1:**
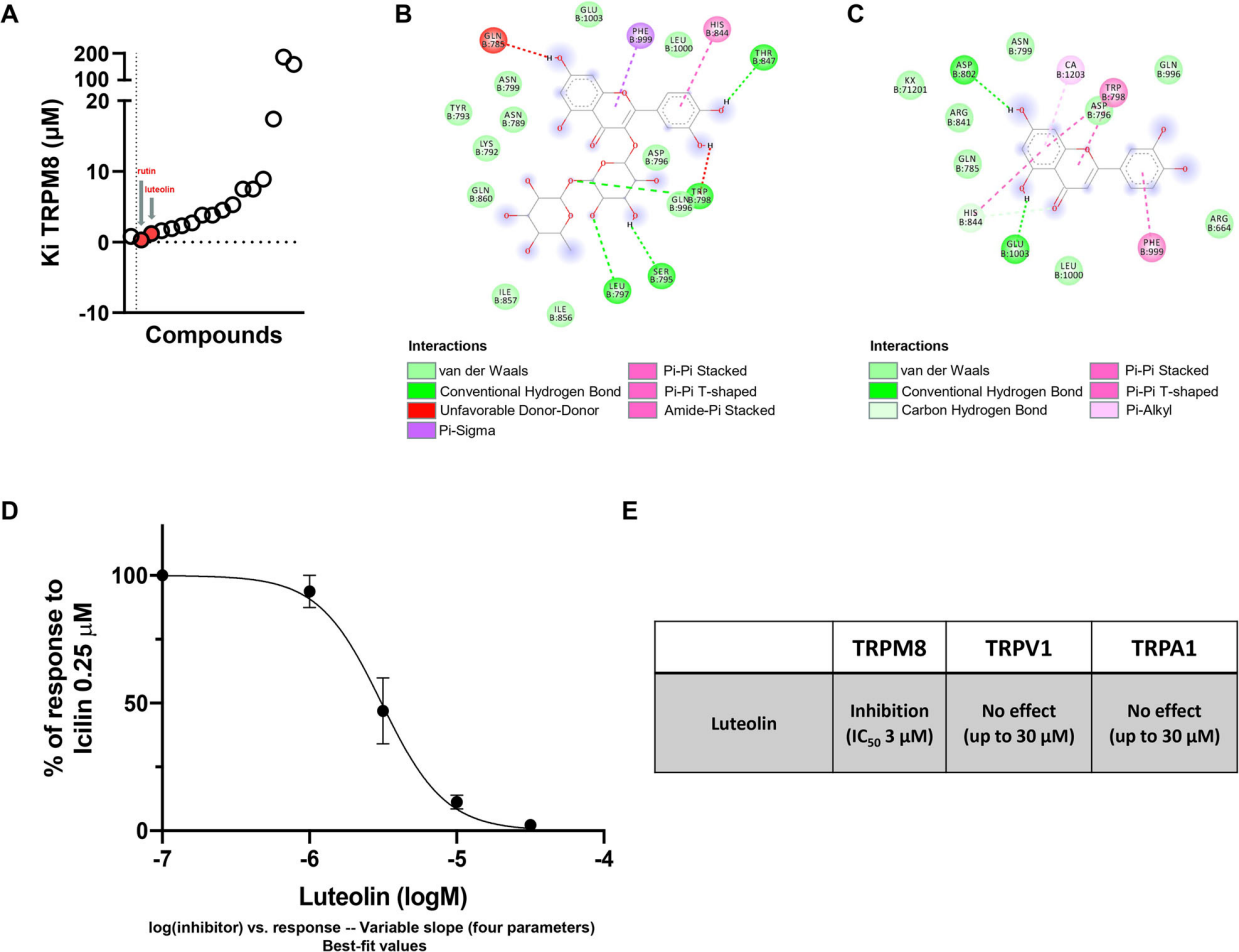
Luteolin is a new selective TRPM8 blocker. From an evaluation of various diet-derived compounds affinity for TRPM8, luteolin emerged as a new selective TRPM8 blocker. **A** Scatter diagram of the theoretically estimated affinity of TRPM8 for 17 diet-derived compounds (Ki). Each point represents a natural compound. In red are reported rutin and luteolin. **B** Rutin structure and binding site on TRPM8. **C** Luteolin structure and binding site on TRPM8. **D** Concentration–response curve of luteolin (0.1–100 μM) on intracellular Ca^2+^ levels in TRPM8- HEK293, measured in the presence of icilin 0.25 μM, the TRPM8 reference agonist. Data represent the mean ± SEM of ≥5 determinations. **E** Effect of luteolin (0.1–30 μM) on intracellular Ca^2+^ levels on HEK293 cells over-expressing TRPM8, TRPV1, and TRPA1 in the presence or not of their respective agonists.

Toward this goal, the potential affinity for the TRPM8 receptor was evaluated using molecular docking simulations within a subset of 17 dietary compounds (Supplementary Table 1) with known anti-inflammatory properties. Menthol and sesamin, a potent natural agonist and antagonist of TRPM8, respectively, were used as references [28, 29] and the affinity for the receptor was reported in terms of the estimated binding affinity (Ki; Fig. 1A). Among the subset of natural compounds, our analysis revealed that rutin and luteolin showed the highest affinity for TRPM8 (Fig. 1B, C). Next, we tested the validity of this in silico analysis by measuring changes of the intracellular Ca^2+^ concentration [Ca^2+^]_i_ in HEK-293 cells stably transfected with a plasmid encoding for the recombinant human TRPM8 protein (denoted hereafter as “TRPM8-HEK293”).

Our analysis revealed that rutin did not induce changes in [Ca^2+^]_i_ in TRPM8-HEK293 cells (data not shown). On the other hand, luteolin antagonized the Ca^2+^ raise evoked by icilin (0.25 μM), a potent selective agonist of TRPM8 [30]. Specifically, icilin-mediated response was inhibited by luteolin (5-min pre-incubation) in a concentration-dependent manner, with an IC50 of 3.091 ± 0.326 μM (Fig. 1D). Notably, luteolin alone did not initiate a TRPM8-mediated Ca^2+^ influx on transfected cells. Non-transfected HEK293 cells were used as control in our experiments.

Next, to evaluate the selectivity of luteolin for TRPM8 channels, the intracellular calcium assay was also performed in HEK293 stably overexpressing recombinant TRPV1 or TRPA1, two different TRP channels highly expressed in the colon [31] and known to be involved in the intestinal inflammation [32, 33]. As shown in Fig. 1E, luteolin did not induce significant effects in TRPV1 or TRPA1 HEK293 cells after testing it both as an agonist and antagonist up to the concentration of 30 μM (Fig. 1E). In summary, these results indicate that luteolin selectively binds and blocks TRPM8 channels.

### Pro-inflammatory stimuli modulate TRPM8 expression and functionality in macrophages

**Fig. 2:**
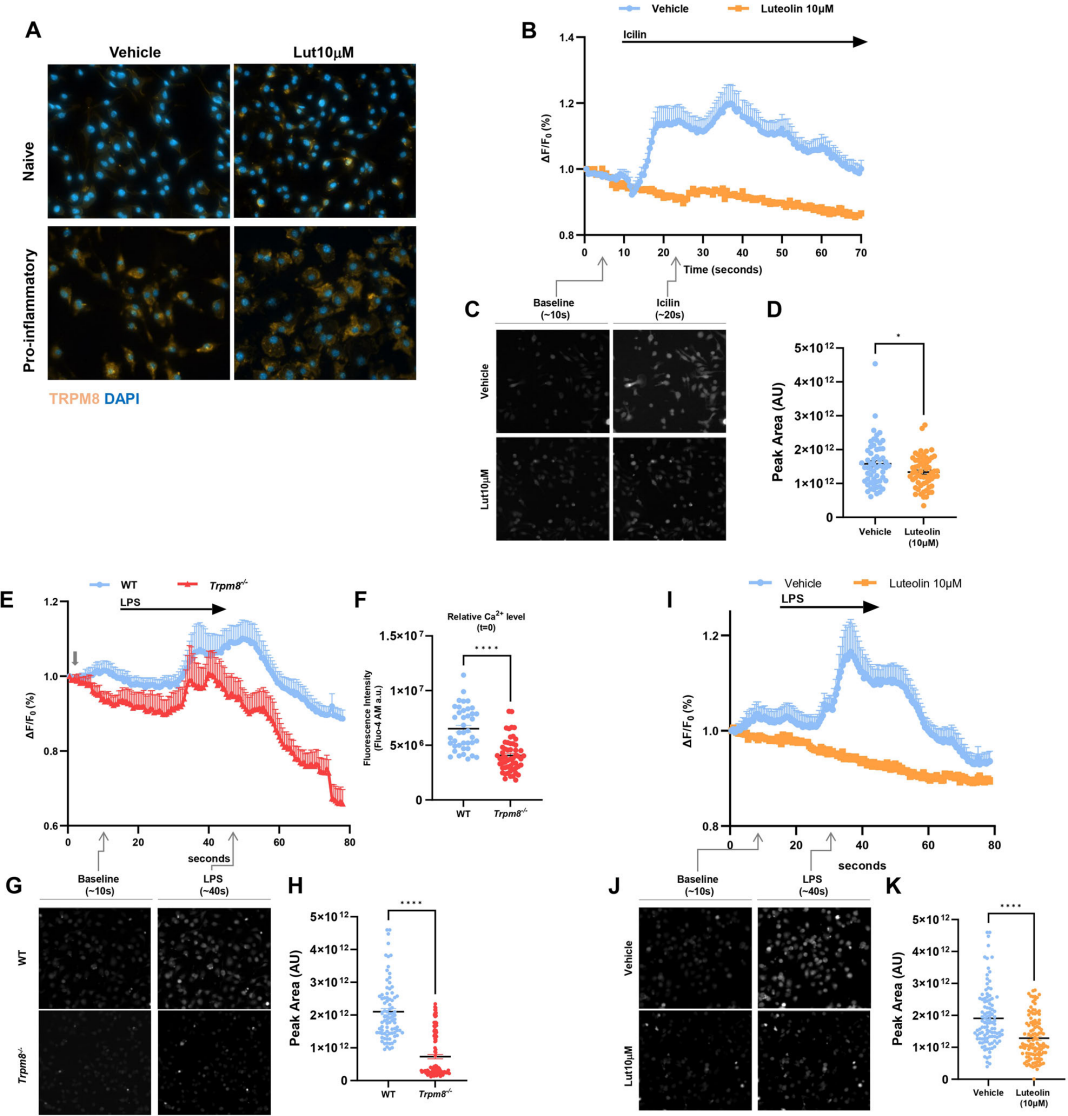
TRPM8 channel activity controls macrophage response to LPS. Over-expression of TRPM8 in pro-inflammatory macrophages is essential for LPS-induced intracellular Ca^2+^ increase. **A** Representative image of TRPM8 (orange), and 4′,6-diamidino-2-phenylindole (DAPI) (blue) immunofluorescence staining in naïve and pro-inflammatory WT BMDMs treated or not with luteolin (10 μM). Analysis was performed 18 h after polarization with LPS + IFN-γ. **B** Relative changes in the fluorescence of Fluo-4AM-loaded naïve BMDMs, reflecting changes in intracellular Ca^2+^ concentration [Ca^2+]^i after icilin (40 μM) perfusion for 1 min. Data are presented as the average of *n* = 3 independent experiments. Error bars represent ±SEM (*n* = 58 and *n* = 60 for Vehicle and Luteolin 10 μM, respectively). **C** Representative fluorescence images from indicated time points in the experiments shown in (**B**). **D** Statistical representation of total peak area of Fluo-4 fluorescence after icilin perfusion from (**B**). Error bars represent ±SEM. *P* value was determined using unpaired Student’s *t*test. *p < 0.05. **E** Relative changes in the fluorescence of Fluo-4AM-loaded naïve WT and *Trpm8*^*−/−*^ BMDMs, reflecting changes in intracellular Ca^2+^ concentration [Ca^2+]^i after LPS (100 ng/mL) perfusion for 30 s. Data are presented as the average of *n* = 3 independent experiments. Error bars represent ±SEM (*n* = 86 and *n* = 112 for WT and *Trpm8*^*−/−*^, respectively). **F** Quantification of basal Ca2+ levels in Fluo-4AM-loaded naïve WT and *Trpm8*^*−/−*^ BMDMs. Error bars represent ±SEM. *P* value was determined using unpaired Student’s *t*test. ****p < 0.0001. **G** Representative fluorescence images from indicated time points in the experiments shown in (**E**). **H** Statistical representation of total peak area of Fluo-4 fluorescence after LPS perfusion from panel E. Error bars represent ±SEM. *P* value was determined using unpaired Student’s *t*-test. ****p < 0.0001. **I** Relative changes in the fluorescence of Fluo-4AM-loaded naïve BMDMs, reflecting changes in intracellular Ca^2+^ concentration [Ca^2+^]i BMDMs after LPS (100 ng/mL) perfusion for 30 s. Data are presented as the average of *n* = 3 independent experiments. Error bars represent ± SEM (*n* = 108 and *n* = 113 for Vehicle and Luteolin 10 μM, respectively). **J** Representative fluorescence images from indicated time points in the experiments shown in (**I**). **K** Statistical representation of total peak area of Fluo-4 fluorescence after LPS perfusion from panel I. Error bars represent ±SEM. *P* value was determined using unpaired Student’s *t* test. ****p < 0.0001.

Activation of macrophages by stimuli, such as LPS and IFN-γ, induces potent pro-inflammatory signaling, strongly depending on the elevation of intracellular Ca^2+^ concentration [34–36]. TRPM8 has been proposed to have an immunomodulatory role in macrophages [25, 26], but the downstream signaling pathways have not been fully elucidated. In this line, we confirmed that TRPM8 expression was enhanced in macrophages upon stimulation with LPS and IFN-γ, compared to naïve cells (Fig. 2A). Consistent with the observed TRPM8 expression in murine BMDMs, we detected large and sustained calcium transients in WT BMDMs in response to the TRPM8 agonist icilin (40 μM) (Fig. 2B–D). In *Trpm8*^*−/−*^ BMDMs, icilin alone was ineffective in increasing the [Ca^2+^]_i_, confirming a TRPM8 selectivity of this compound at the tested concentration (Supplementary Fig. 1). To further confirm the inhibitory effect of luteolin on TRPM8, we pre-incubated BMDMs in the presence (or absence) of this flavonoid (10 μM) for 10 min before the perfusion with icilin (40 μM, Fig. 1). Considering that luteolin was cytotoxic starting from the concentration of 30 µM on both primary (Supplementary Fig. 2) and immortalized (i.e., RAW264.7, Supplementary Fig. 3) macrophages, higher concentrations than 10 μM were not used in our experiments. Importantly, 10 min pre-incubation with luteolin antagonized the effect of icilin in WT BMDMs (Fig. 2B–D), further demonstrating that this flavonoid blocks TRPM8-mediated Ca^2+^ influx.

To further strengthen the role of TRPM8 in the pro-inflammatory function of BMDMs, we performed an epigenetic analysis to evaluate the methylation status of *Trpm8* chromatin in naïve, and pro-inflammatory macrophages, treated or not with luteolin. We used specific primer pairs to search for differences in the methylation vs unmethylation status of *Trpm8* chromatin in a CpG site-rich region identified 2200 bp upstream of the ATG of the first exon. Our analysis revealed that in the pro-inflammatory state, the signal generated by primers designed for an unmethylated status of *Trpm8* chromatin was tendentially stronger than the one generated by methylated ones (Supplementary Fig. 1), possibly indicating that the transcription of the gene is activated by the pro-inflammatory condition. Remarkably, when compared to untreated cells, in luteolin-treated pro-inflammatory macrophages, the DNA amplification signal generated by unmethylated UF2-UR2 and UF3-UR3 primers was reduced (Supplementary Fig. 1), suggesting that the regulation of *Trpm8* expression is one of the mechanisms underlying the anti-inflammatory effects of luteolin.

### TRPM8 is required for LPS-induced Ca^2+^ influx in macrophages

In macrophages LPS initiates a rapid rise in intracellular Ca^2+^ levels [37], however, the underlying mechanism remains largely unclear. Considering that TRPM8 can mediate Ca^2+^ responses in macrophages and is upregulated upon stimulation with LPS in our experimental conditions (Fig. 2A–D), we hypothesized that TRPM8 mediates the Ca^2+^ currents evoked by LPS. To test this, we measured LPS-induced intracellular Ca^2+^ changes in WT or *Trpm8*^*−/−*^ BMDMs. In line with previous reports, WT macrophages displayed a robust rise in cytosolic calcium levels after LPS (100 ng/mL) stimulation (Fig. 2E–H). In sharp contrast, *Trpm8*^*−/−*^ BMDMs showed significantly lower levels of basal fluorescence intensity and were weakly responsive to LPS (Fig. 2F, H). On the other hand, no differences were detected between WT and *Trpm8*^*−/−*^ BMDMs in response to ATP (Supplementary Fig. 1).

In line with these findings, the pharmacological inhibition of TRPM8 in WT BMDMs by 10 min pre-incubation with luteolin blocked LPS-induced Ca^2+^ influx (Fig. 2I–K). Luteolin effect was mirrored by pre-incubation with AMTB (10 min; Supplementary Fig. 1), a well-known specific TRPM8 antagonist [38, 39], strongly supporting the role of TRPM8 channel in luteolin pharmacological activity. Interestingly, both luteolin and AMTB treatment reduced the fluorescence level of basal Ca^2+^ indicator in the naïve cells before LPS stimulation (Supplementary Fig. 1).

Taken together, these results indicate that TRPM8 channels have constitutive activity, contributing to resting [Ca^2+^]_i_ in BMDMs, and mediating the increase in intracellular Ca^2+^ induced by LPS.

### TRPM8 deletion or pharmacological targeting by luteolin reduces macrophage pro-inflammatory capacity

**Fig. 3:**
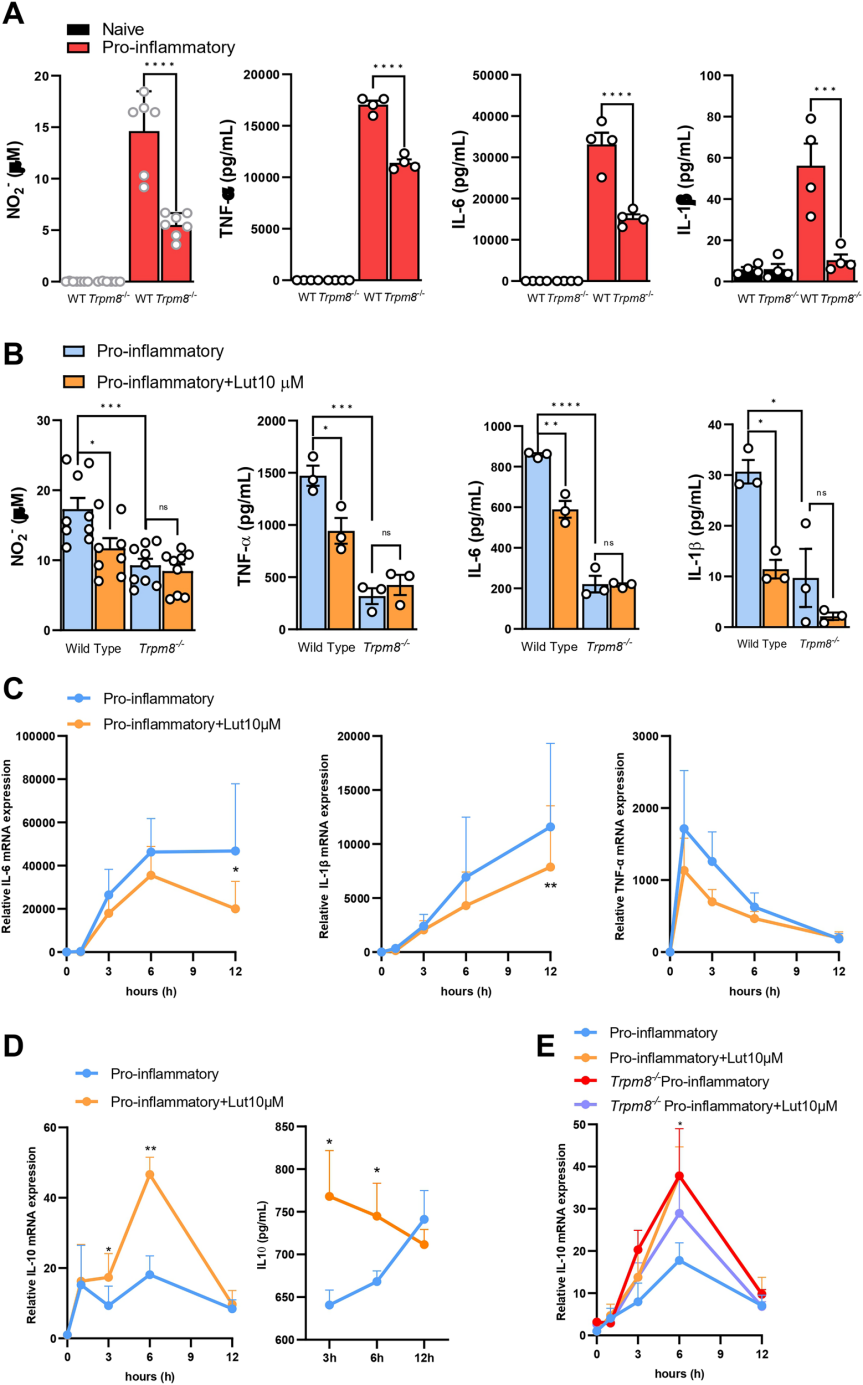
TRPM8 is required for pro-inflammatory polarization of macrophages. TRPM8 blockade with luteolin, or its genetic ablation, blunt macrophage pro-inflammatory capacity. **A** Nitrite levels and TNF-α, IL-6, and IL-1β levels were measured in the supernatant of naïve and pro-inflammatory WT or *Trpm8*^*−/−*^ BMDMs. Analysis was performed 18 h after polarization with LPS + IFN-γ. Data are presented as the average of n ≥ 3 independent experiments, with each dot representing an individual mouse. Error bars represent mean ± SEM. P value was determined using Two-way ANOVA followed by Šídák’s multiple comparisons test. ***p < 0.001; ****p < 0.0001. **B** Nitrite levels and TNF-α, IL-6, and IL-1β levels were measured in the supernatant of pro-inflammatory WT or *Trpm8*^*−/−*^ BMDMs treated or not with luteolin (10 μM). Analysis was performed 18 h after polarization with LPS + IFN-γ. Data are presented as the average of *n* ≥ 3 independent experiments, with each dot representing an individual mouse. Error bars represent ±SEM. *P* value was determined using two-way ANOVA followed by Šídák’s multiple comparisons test. ns non significant,*p < 0.05; **p < 0.01; ***p < 0.001; ****p < 0.0001. **C** IL-6, IL-1β, and TNF-α mRNA relative expression in pro-inflammatory WT BMDMs treated or not with luteolin (10 μM). The analysis was performed after 0, 1, 3, 6, or 12 h of polarization with LPS + IFN-γ. Data are expressed as relative to basal levels (0 h timepoint) and are presented as the average of *n* = 5 independent experiments. Error bars represent ±SEM. *P* value was determined using multiple paired Student’s *t*test. *p < 0.05; **p < 0.01. **D** IL-10 relative mRNA levels (left) and protein levels (right) measured in pro-inflammatory WT BMDMs treated or not with luteolin (10 μM). The analysis was performed after 0, 1, 3, 6, or 12 h of polarization with LPS + IFN-γ. mRNA data are expressed as relative to basal levels (0 h timepoint). All the data are presented as the average of *n* ≥ 5 independent experiments. Error bars represent ±SEM. *P* value was determined using paired multiple Student’s *t* test. *p < 0.05; **p < 0.01. **E** IL-10 relative mRNA levels measured in pro-inflammatory WT or *Trpm8*^*−/−*^ BMDMs treated or not with luteolin (10 μM). The analysis was performed after 0, 1, 3, 6, or 12 h of polarization with LPS + IFN-γ. Data are expressed as relative to basal levels (0 h timepoint) and are presented as the average of *n* = 5 independent experiments. Error bars represent ±SEM. *P* value was determined using multiple paired Student’s *t* test. *p < 0.05.

To further understand the contribution of these channel receptors to the macrophage pro-inflammatory capacity, we assessed the effect of TRPM8 genetic ablation as well as the consequences of TRPM8 blockage by luteolin pre-treatment on the production of nitrites, IL-1β, IL-6 and TNF-α in BMDMs (Fig. 3). Whereas LPS and IFN-γ treatment significantly increased the release of nitrites, IL-1β, IL-6, and TNF-α in WT BMDMs, *Trpm8*^*−/−*^ BMDMs had a blunted response and an overall decreased expression of all the aforementioned pro-inflammatory mediators (Fig. 3A). These data strongly support the role of TRPM8 in determining a pro-inflammatory phenotype in macrophages.

Accordingly, pre-treatment with luteolin 1 h before LPS stimulation reduced nitrite production in a concentration-dependent manner (Supplementary Fig. 3) in immortalized pro-inflammatory macrophages (i.e., RAW264.7), a cell line known to express TRPM8 [25]. Among the concentrations tested, the 10 μM was the most effective, reducing nitrite production by 77.15% compared to vehicle-treated pro-inflammatory cells. Therefore, we further used 10 μM luteolin in all the following experiments on BMDMs. Luteolin (10 μM) significantly reduced nitrite production and pro-inflammatory cytokine release in WT BMDMs compared to untreated cells (Fig. 3B). Of note, luteolin did not affect *Trpm8*^*−/−*^ BMDMs (Fig. 3B), confirming that its anti-inflammatory effect is TRPM8-mediated.

To shed light on luteolin downstream molecular mechanism, we measured the pro-inflammatory cytokine expression in WT BMDMs at different time points (i.e., 1, 3, 6, and 12 h after polarization with LPS + IFN-γ). When compared to untreated cells, luteolin (10 μM) significantly reduced IL-6 and IL-1β gene expression after 6–12 h, with a decreasing trend also observed in TNF-α gene levels (Fig. 3C). Worthy of note, blocking TRPM8 via luteolin treatment significantly increased both gene and protein levels of IL-10 in WT macrophages at very early timepoints (i.e., 3–6 h after polarization with LPS + IFN-γ) (Fig. 3D). Additionally, luteolin significantly increased the early expression of *Mrc1* and showed a trend toward increasing *Arg1* at later timepoints (Supplementary Fig. 4), suggesting a shift in the polarization of pro-inflammatory macrophages toward a pro-resolving phenotype. In line, LPS + IFN-γ stimulated *Trpm8*^*−/−*^ BMDMs showed higher levels of IL-10 at early timepoints (Fig. 3E), supporting the hypothesis that TRPM8 inhibition can regulate pro-tolerogenic gene expression in macrophages.

### TRPM8 controls the metabolic status of pro-inflammatory macrophages

**Fig. 4:**
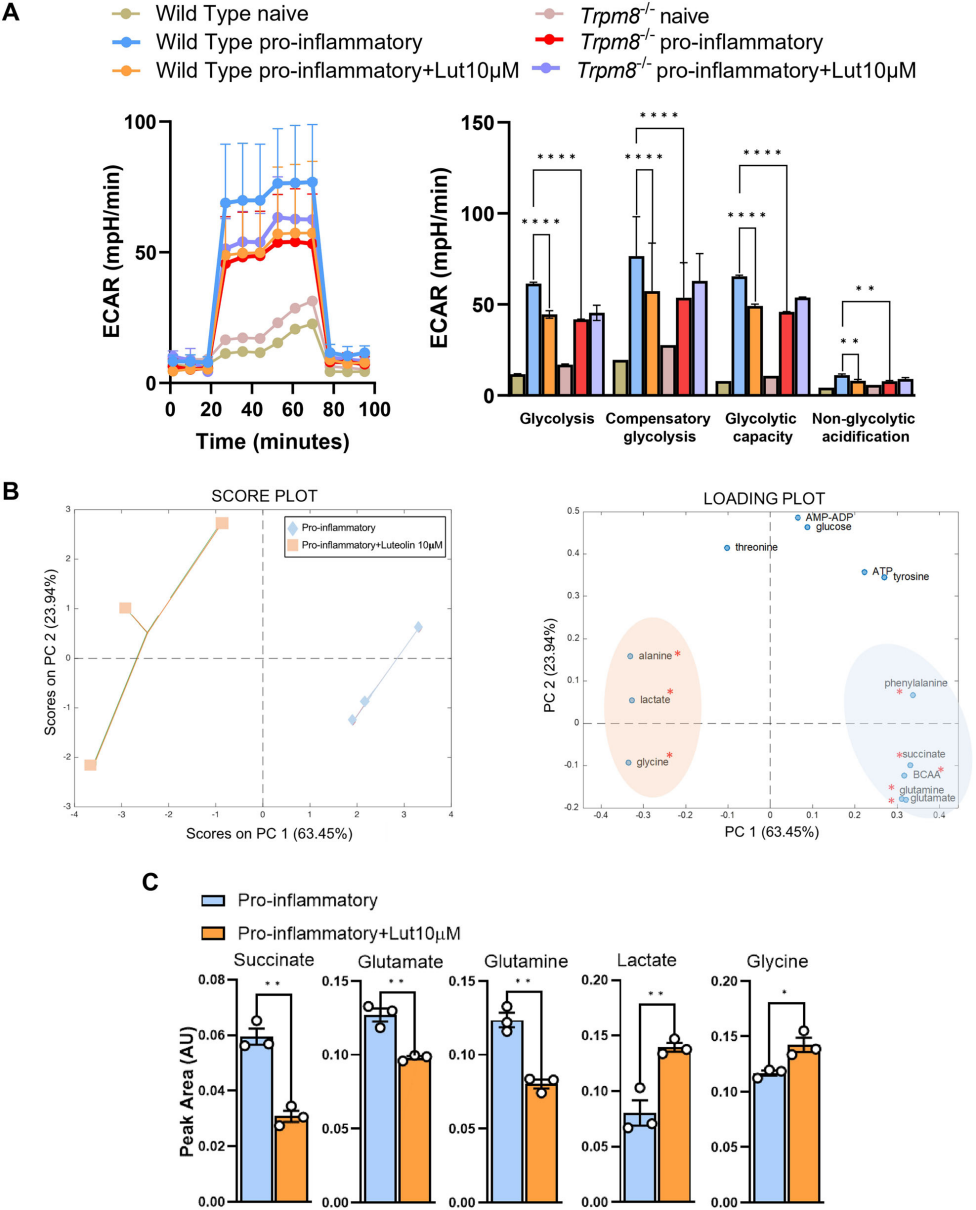
TRPM8 regulates immunometabolic pathways in macrophages. TRPM8 modulation with luteolin reduces the metabolic rewiring of macrophages, affecting the levels of the main immunoregulatory metabolites. **A** ECAR kinetics (left) and glycolytic parameters (right) of naïve and pro-inflammatory WT and Trpm8^−/−^ BMDMs treated or not with Luteolin (10 μM). Analysis was performed 18 h after polarization with LPS + IFN-γ. Data are presented as the average of *n* = 3 independent experiments. Error bars represent ±SEM. *P* value was determined using one-way ANOVA followed by Tukey’s multiple comparisons test. **p < 0.01; ****p < 0.0001. Score and loading plots derived from the PCA model (**B**) and succinate, lactate, glycine, glutamine, and glutamate levels (**C**) measured by NMR metabolomic analysis of pro-inflammatory RAW 264.7 treated or not with Luteolin (10 μM). Analysis was performed 18 h after polarization with LPS. **C** Data are presented as peak area normalized on total area of n = 3 independent experiments. Error bars represent ±SEM. *P* value was determined using unpaired Student’s *t* test. *p < 0.05; **p < 0.01.

Over the past decade, it has been recognized that the profile of cellular metabolism plays a pivotal role in macrophage activation, with early IL-10 production being the primary anti-inflammatory mechanism that controls the metabolic switch triggered in macrophages by pro-inflammatory stimuli [40]. Therefore, we aimed to investigate whether luteolin could reduce the pro-inflammatory capacity of macrophages through an IL-10-mediated effect on their metabolic remodeling. Thus, we first determined the extracellular acidification rate (ECAR) in WT BMDMs stimulated with LPS and IFN-γ, in the presence or not of luteolin (10 μM) for 18 h. LPS and IFN-γ stimulation increased the ECAR in WT BMDMs as previously reported [41] (Fig. 4A). Luteolin treatment significantly reduced both basal and compensatory glycolysis, glycolytic capacity, and non-glycolytic acidification in WT, but not in *Trpm8*^*-/-*^ macrophages (Fig. 4A). Accordingly, *Trpm8*^*−/−*^ macrophages stimulated with LPS + IFN-γ showed a significantly reduced ECAR compared to WT BMDMs (Fig. 4A). Overall, these results prove that TRPM8 sustains pro-inflammatory macrophage activation through a specific effect on glycolytic metabolism.

To further define the luteolin effect on the metabolic status of macrophages, we performed a ^1^H-NMR-based metabolomic analysis. Due to the complexity and heterogeneity of BMDMs, we initially performed an untargeted analysis on the lysates of LPS-stimulated immortalized macrophages (i.e., RAW264.7) treated or not with luteolin (10 μM) for 18 h. Firstly, we assessed the metabolic effect of luteolin (10 μM) in RAW264.7 cells. Consistent with our findings (Fig. 4A), luteolin treatment significantly reduced ECAR, as well as both basal and compensatory glycolysis, in LPS-stimulated cells (Supplementary Fig. 3). Thus, a principal component analysis (PCA) was performed to explore the differences in cell metabolome under the different experimental conditions. Interestingly, a clear separation occurred between untreated and luteolin-treated pro-inflammatory macrophages, mirroring a metabolic switch induced by luteolin (Fig. 4B). These differences were driven by a differential expression of amino acids (i.e., glutamate, glutamine, glycine), but also succinate and lactate levels (Fig. 4B). Specifically, LPS + IFN-γ polarization induced a significant increase in succinate, glutamate, and glutamine in macrophages, whereas luteolin treatment was sufficient to prevent their increment (Fig. 4C). The enhanced production of succinate and glutamate is a critical regulator of the pro-inflammatory response through the inhibition of anti-inflammatory genes, while glutamate and glutamine metabolism is known to support the anti-inflammatory polarization of macrophages [42–44]. Interestingly, glutamine can be metabolized into lactate in tumor cells and macrophages [45], and luteolin-treated macrophages exhibited significantly higher levels of lactate compared to untreated pro-inflammatory BMDMs (Fig. 4C). Finally, luteolin treatment also upregulated the levels of glycine, the final product of the pentose phosphate pathway, responsible for macrophage polarization towards a pro-resolutive phenotype [46].

### Lactate production is required for IL-10-mediated functional reprogramming in luteolin-treated macrophages

**Fig. 5:**
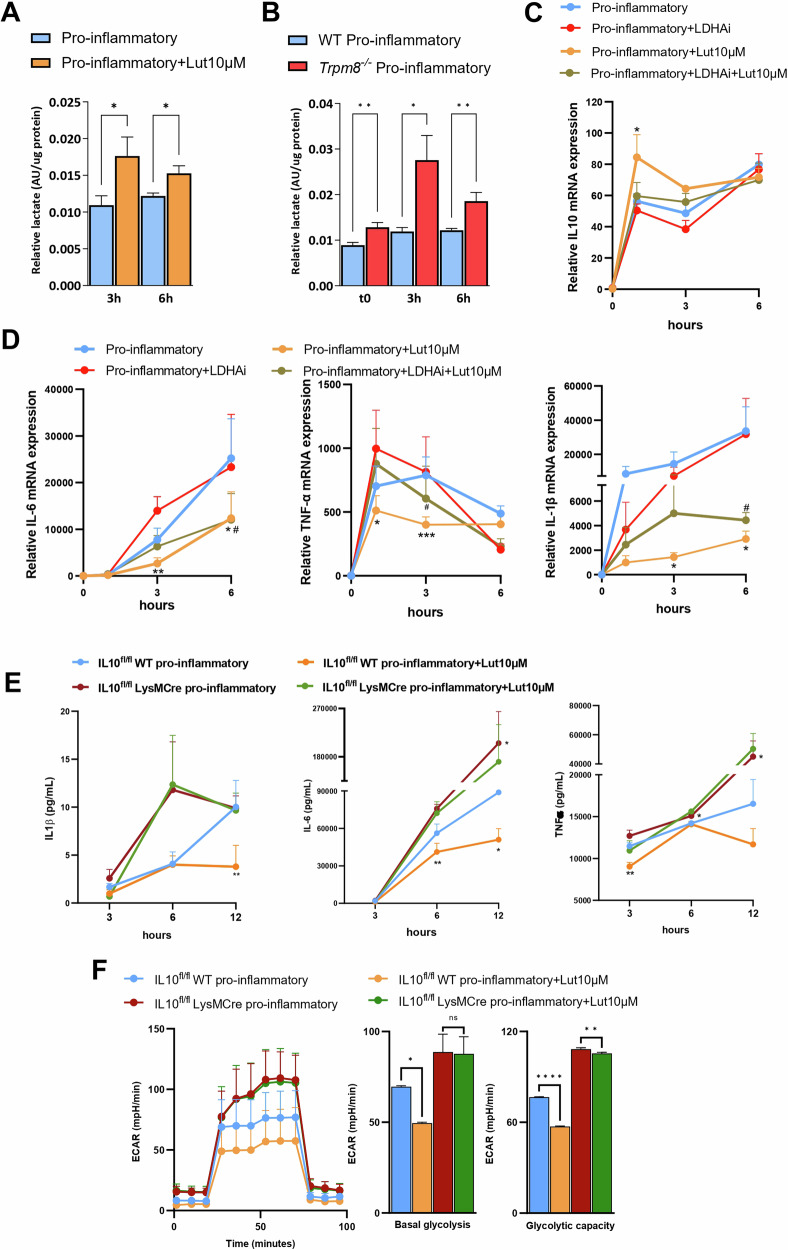
Blocking of TRPM8 induces lactate-mediated activation of IL-10 pathway. Luteolin, via TRPM8, increases lactate levels necessary for IL-10 pathway activation. **A** Relative lactate levels measured by GC-MS metabolomic analysis of pro-inflammatory BMDMs treated or not with Luteolin (10 μM). The analysis was performed after 3 or 6 h of polarization with LPS + IFN-γ. Data are presented as peak area normalized on μg of protein of *n* = 3 independent experiments, each performed in technical triplicate. Error bars represent ±SEM. *P* value was determined using unpaired Student’s *t* test. *p < 0.05. **B** Relative lactate levels measured by GC-MS metabolomic analysis of WT and *Trpm8*^*−/−*^ BMDMs. The analysis was performed in basal conditions (t0) and after 3 or 6 h of polarization with LPS + IFN-γ. Data are presented as peak area normalized on μg of protein of *n* = 3 independent experiments, each performed in technical triplicate. Error bars represent ± SEM. *P* value was determined using unpaired Student’s *t* test. *p < 0.05; **p < 0.01. IL-10 (**C**) and IL-6, TNF-α, and IL-1β (**D**) mRNA relative expression measured in pro-inflammatory WT BMDMs treated or not with luteolin (10 μM) and LDH-a inhibitor GSK2837808A (10 μM). The analysis was performed after 0, 1, 3, or 6 h of polarization with LPS + IFN-γ. Data are expressed as relative to basal levels (0 h timepoint) and are presented as the average of *n* = 5 independent experiments. Error bars represent ±SEM. *P* value was determined using multiple paired Student’s *t* test. *p < 0.05; **p < 0.01; ***p < 0.001 Pro-inflammatory+Lut10μM *vs* Pro-inflammatory; #p < 0.05 Pro-inflammatory+LDHAi+Lut10μM *vs* Pro-inflammatory+LDHAi. **E** IL-6, IL-1β, and TNF-α levels measured in the supernatant of *IL-10*^fl/fl^ WT and *IL-10*^fl/fl^ LysMCre BMDMs treated or not with luteolin (10 μM), after 3, 6, or 12 h of polarization with LPS + IFN-γ. Data are presented as the average of *n* = 5 independent experiments. Error bars represent ±SEM. *P* value was determined using multiple paired Student’s *t* test. *p < 0.05; ** *p* < 0.01 *vs IL-10*^fl/fl^ WT pro-inflammatory. **F** ECAR kinetics (left) and glycolytic parameters (right) of naïve and pro-inflammatory *IL-10*^fl/fl^ WT and *IL-10*^fl/fl^ LysMCre BMDMs treated or not with Luteolin (10 μM). Analysis was performed 18 h after polarization with LPS + IFN-γ. Data are presented as the average of *n* = 3 independent experiments. Error bars represent ±SEM. P value was determined using one-way ANOVA followed by Tukey’s multiple comparisons test. n.s. non-significant, *p < 0.05; **p < 0.01; ****p < 0.0001.

Recently, lactate was shown to significantly delay the upregulation of the majority of LPS-induced genes in macrophages and promote the expression of genes deputed to tissue repair [47, 48]. We, therefore, hypothesized that luteolin mechanism of action may be partially mediated by an increase in intracellular lactate levels at early timepoints, possibly leading to the induction of tissue repair genes, such as IL-10. To verify this hypothesis, we examined the dynamics of lactate production during BMDMs polarization after the stimulation with LPS and IFN-γ, in the presence or absence of luteolin (10 μM). We observed that luteolin-treated WT BMDMs exhibited a significant increase in lactate levels starting from 3 h after stimulation with LPS + IFN-γ (Fig. 5A). In line with these results, luteolin treatment also reduced the levels of pyruvate, the primary lactate source in macrophages, at early timepoints (Supplementary Fig. 4). To prove whether luteolin effect on the biosynthetic pathway of lactate is also mediated by TRPM8, we analyzed the production of such metabolites in *Trpm8*^*−/−*^ BMDMs. Genetic deletion of TRPM8 in macrophages induced a significant increase of lactate concentration in basal conditions (t0), 3 and 6 h after LPS + IFN-γ (Fig. 5B), confirming that the lack of TRPM8 mirrors the effect of its pharmacological inhibition.

The reduction of pyruvate to lactate is catalyzed by lactate dehydrogenase (LDH), a rate-limiting enzyme for lactate production. Therefore, to corroborate our hypothesis, we pharmacologically blocked lactate dehydrogenase A (LDHa) by using the selective inhibitor GSK2837808A [49]. Following a 12 h incubation period with the LDHa inhibitor (10 μM), we polarized naïve wild-type BMDMs towards a pro-inflammatory phenotype in the presence or not of luteolin. As expected, GSK2837808A significantly reduced the lactate production in wild-type BMDMs (Supplementary Fig. 4). Due to decreased lactate levels at early timepoints, luteolin (10 µM) failed to boost IL-10 production in pro-inflammatory macrophages treated with the LDHa inhibitor (Fig. 5C). Further, lactate deprivation in LPS and IFN-γ stimulated macrophages significantly impaired the anti-inflammatory effect of luteolin in reducing IL-6, TNF-α and IL-1β gene expression (Fig. 5D). However, the anti-inflammatory effect was regained over time (Fig. 5D). Together, these results indicate that lactate generated by LDHa activity is crucial for supporting the early phase of the anti-inflammatory effect of luteolin.

To finally determine whether the increase in IL-10 production is responsible for luteolin anti-inflammatory and metabolic effects in macrophages, we used BMDMs derived from IL-10^fl/fl^ LysMCre mice. As expected, IL-10 levels were ablated in LysMcre mice (IL-10^fl/fl^ LysMCre, Supplementary Fig. 4), and, in accordance with the literature [40], they presented significantly higher levels of IL-6 and TNF-α, and a notable but non-significant increase in IL-1β levels compared to their control counterpart (IL-10^fl/fl^ WT) (Fig. 5E). Notably, luteolin treatment significantly reduced the pro-inflammatory cytokines release in IL-10^fl/fl^ WT macrophages but was ineffective in LysMCre macrophages (Fig. 5E). These data were strongly supported by the reduction of luteolin effect on the metabolic rewiring induced by LPS and IFN-γ stimulation in IL-10 deprived macrophages (Fig. 5F).

Overall, these results clearly demonstrate that lactate increase upon luteolin treatment is essential for its immunomodulatory effect, through the upregulation of the IL-10 pathway.

### Luteolin reduces colitis severity and lower recruitment of inflammatory myeloid cells in the gut

**Fig. 6:**
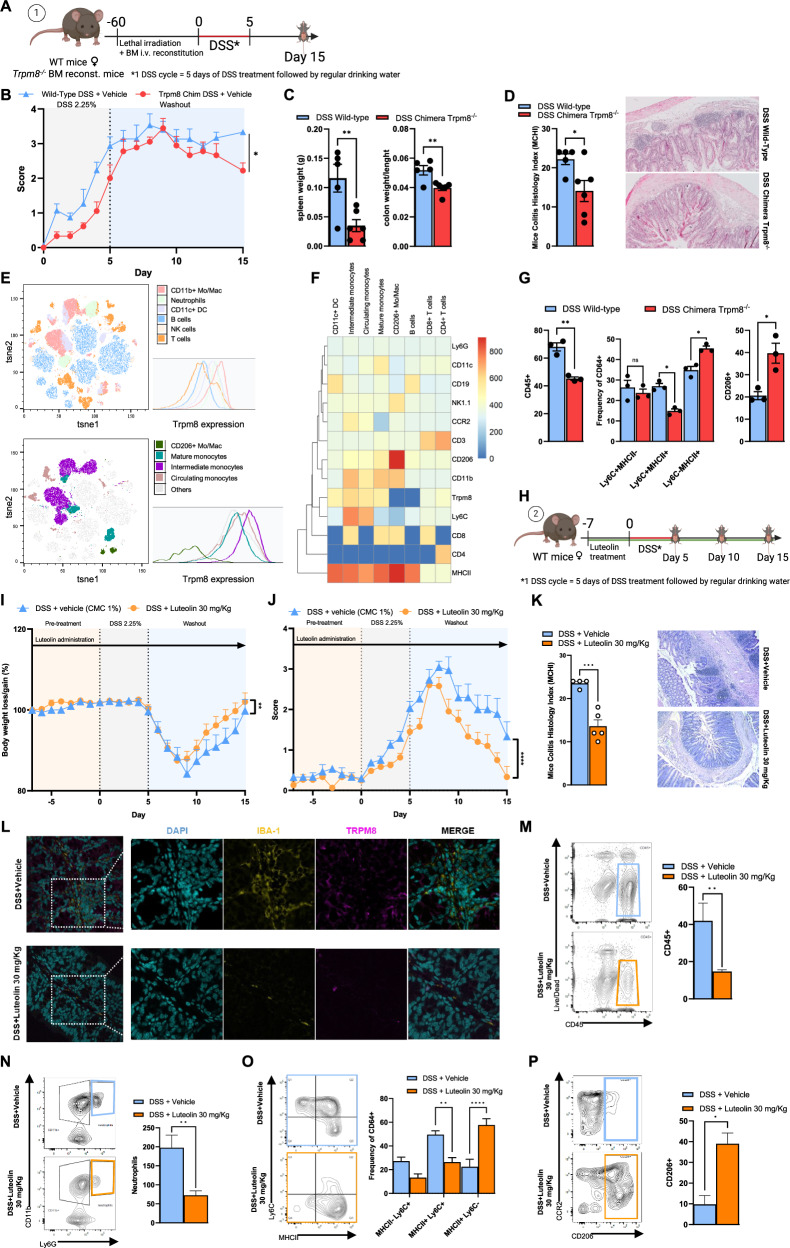
Blocking TRPM8 in mice ameliorates DSS-induced colitis. WT mice reconstituted with *Trpm8*^*−/−*^ BM cells or supplemented with oral luteolin are resistant to DSS-induced colitis by reduced recruitment of inflammatory myeloid cells. **A** Schematic representation of DSS protocol and timeline. **B** Disease activity index (DAI) of colitis severity. Data are presented as the average of *n* = 5 mice for each experimental group. Error bars represent ±SEM. *P* value was determined using Student’s *t* test. *p < 0.05. **C** Spleen weight (g) and weight/length ratio (mg/cm) of colon tissue in WT mice reconstituted or not with *Trpm8*^*−/−*^ BM. Error bars represent ±SEM. *P* value was determined using unpaired Student’s t-test. **p < 0.01. **D** Histological score (left) and representative H&E images (right) of colon sections of WT mice reconstituted or not with *Trpm8*^*−/−*^ BM. Error bars represent mean ± SEM. *P* value was determined using an unpaired Student’s *t* test. *p < 0.05. **E** tSNE + FlowSOM analysis performed on CD45^+^ lineage of lamina propria of colon samples from WT mice subjected to DSS administration (*n* = 3 mice). **F** Heatmap representing Trpm8 expression throughout the clusters of CD45^+^ lineage from lamina propria of colon samples from WT mice subjected to DSS administration (*n* = 3 mice). **G** Expression of CD45, Ly6C, MHCII and CD206 by cells isolated from colonic lamina propria of WT mice reconstituted or not with *Trpm8*^*−/−*^ BM. Data are presented as the average of *n* = 3 mice for each experimental group. Error bars represent ±SEM. *P* value was determined using unpaired Student’s *t* test. ns non-significant; *p < 0.05; **p < 0.01. **H** Schematic representation of DSS protocol and timeline. **I** Mice body weight gain/loss. Data are presented as the average of *n* = 10 mice for each experimental group. Error bars represent ±SEM. P value was determined using unpaired Student’s *t* test. **p < 0.01. **J** Disease activity index (DAI) of colitis severity. Data are presented as the average of *n* = 10 mice for each experimental group. Error bars represent ±SEM. *P* value was determined using Student’s *t* test. ****p < 0.0001. **K** Histological score (left) and representative H&E images (right) of mice colon sections. Error bars represent mean ± SEM. *P* value was determined using an unpaired Student’s *t* test. ***p < 0.001. **L** Representative confocal images of TRPM8 (magenta), IBA-1 (yellow), and DAPI (cyan) immunostaining in DSS+Vehicle or DSS+Luteolin treated mice, collected 15 dpt. Analyses were carried out on five mice for each experimental group. Representative flow plots and count of CD45 (**M**) and neutrophils (**N**) populations in lamina propria of mice colon. Data are presented as the average of *n* = 5 mice for each experimental group. Error bars represent ±SEM. *P* value was determined using unpaired Student’s *t* test. **p < 0.01. Representative flow plots and expression of Ly6C and MHCII (**O**) and CCR2 and CD206 (**P**) by monocytes isolated from lamina propria of mice colon. Data are presented as the average of *n* = 5 mice for each experimental group. Error bars represent ±SEM. *P* value was determined using unpaired Student’s t-test. *p < 0.05; **p < 0.01; ****p < 0.0001.

To explore the pathophysiological significance of our findings in the context of intestinal inflammation, we aimed to evaluate the role of TRPM8 in immune function in vivo by using a bone marrow transplant model. WT mice were lethally irradiated (9.5 Gy) and subsequently reconstituted intravenous (i.v.) with *Trpm8*^*−/−*^ or WT bone marrow (BM). A schematic of the experimental protocol/timeline is given in Fig. 6A. Mice were then subjected to dextran sulfate sodium (DSS) model of colitis (2.25% DSS in drinking water for 5 days; Fig. 6A). WT mice reconstituted with *Trpm8*^*−/−*^ BM was significantly more resistant to colitis, showing a reduced disease activity index (DAI; Fig. 6B), reduced spleen weight and colon weight to length ratio (Fig. 6C). This reduction in colitis severity was accompanied by improvement in colonic histological score in *Trpm8*^*−/−*^ chimera mice, which showed a significant reduction in goblet cell loss, crypt density hyperplasia, and leukocyte infiltration compared to the WT group (Fig. 6D). In an effort to dissect the immune populations responsible for this effect, we characterized the composition of colonic lamina propria leukocytes by flow cytometry. The analysis showed that among all the immune populations, TRPM8 was mainly expressed by myeloid cells (CD11b^+^, Ly6C^+^, MHCII^+^) (Fig. 6E). Most interestingly, the analysis of the CD11b^+^ population showed that Trpm8 expression was higher in Ly6C^+^ monocytes and Ly6C^+^MHCII^+^ intermediate monocyte-derived macrophages, while it was downregulated by mature MHCII^+^ resident-like and not expressed by CD206^+^ macrophages (Fig. 6F). Accordingly, WT-*Trpm8*^*−/−*^ BM chimera mice showed a reduction of CD45^+^ immune cells in the colonic lamina propria, and more specifically, a significant reduction in the number of Ly6C^hi^MHCII^+^ monocyte-derived macrophages paired to a significant increase of mature Ly6C^-^MHCII^+^ and of CD206^+^ macrophages (Fig. 6G).

Further, we also aimed to evaluate the anti-inflammatory effect of luteolin in the DSS model of colitis. Even in this case, WT mice were exposed to 2.25% DSS in drinking water for 5 days (Fig. 6H) and treated by oral gavage with luteolin at the doses of 3, 10, and 30 mg/kg for 21 days (Supplementary Fig. 5). This range of doses was chosen accordingly to previous studies [50]. Compared to vehicle-treated mice, luteolin (30 mg/kg) counteracted the DSS-induced weight loss (Fig. 6I), and reduced the DAI, showing earlier recovery of symptoms (Fig. 6J). Once again, a significant improvement in colonic histological score was also observed following luteolin treatment (Fig. 6K). Importantly, luteolin treatment had no impact on gut homeostasis in healthy mice (Supplementary Fig. 5).

Confocal immunofluorescence revealed that luteolin-treated mice subjected to DSS clearly had a reduction in macrophage infiltration and a lower expression of Trpm8, compared to the vehicle (Fig. 6L). In line with our flow cytometry data, immunofluorescence of the colonic mucosa indicated that Trpm8 was co-localized with Iba-1 in vehicle-treated mice, (Fig. 6E, L). Accordingly, ex-vivo experiments on murine colon stimulated with LPS revealed that luteolin (10 μM) significantly reduced the expression of inducible nitric oxide synthase (iNOS) and of the main pro-inflammatory cytokines released by activated macrophages (i.e., IL-6, IL-1b, and TNF-α), further underlying the importance of TRPM8 modulation on macrophages in the gut (Supplementary Fig. 6).

We next aimed to study the effect of luteolin on the immune cells during colitis. Neutrophils, eosinophils, monocytes, immature macrophages, and mature CD206^+^ macrophages from the colonic lamina propria of mice were studied and characterized by flow cytometry, 5-, 10- or 15-days post-treatment (dpt) (Fig. 6M–P, Supplementary Fig. 5). Flow cytometric analysis revealed that luteolin treatment did not significantly alter the immune response during the acute phase of DSS colitis (Supplementary Fig. 5). However, a reduced absolute number of immune cells in the colonic lamina propria of luteolin-treated mice was observed during the recovery phase after induction of colitis compared with the vehicle group, mirroring the effect of TRPM8 conditional immune genetic deletion (Fig. 6M). Luteolin oral administration induced a significant reduction in the number of Ly6G^+^ neutrophils (Fig. 6N) and Ly6C^hi^ monocytes and immature macrophages (Fig. 6O), and a significant increase of mature Ly6C^-^MHCII^+^ and CD206+ macrophages (Fig. 6O, P) during DSS colitis.

Taken together these data indicate that TRPM8 deletion from immune cells in mice, as well as luteolin supplementation, counteracts intestinal inflammation and mediates an impairment in the innate immune response during experimental colitis.

## Discussion

In this study, we provide new insights into the crucial immunomodulatory role of TRPM8 in macrophages and identify luteolin, a flavonoid found in various vegetables and fruit [51], as its novel potent inhibitor. By elucidating the target and molecular pathway underlying luteolin anti-inflammatory effects, we shed light on a previously unknown mechanism supporting its therapeutic potential. Here, we show that luteolin antagonized the Ca^2+^ elevation response to icilin in TRPM8-HEK293 cells, without activating or inhibiting TRPV1- or TRPA1, the main TRP channels involved in gut inflammation [32]. Although the main function of TRPM8 has been historically indicated as a cold sensor in peripheral sensory neurons [52], growing evidence accumulated in the past few years points to additional functions. While prominently expressed in a proportion of sensory fibers, TRPM8 is also found in other cell types, including murine innate immune cells and human monocytes, where it has been shown to play a role in their differentiation in response to inflammatory stimuli [25, 26]. Elevated TRPM8 levels are observed in inflamed murine and human colons [23], indicating that channel activation may be required during the inflammatory process. However, its specific role in chronic inflammatory conditions like IBD remains contradictory and requires further mechanistical investigation [23–25]. Previous works show conflicting results, with some studies indicating more severe inflammation in Trpm8-deficient mice during DSS colitis [24, 25], while other researchers suggest otherwise [23]. Here, we observe an increase in *Trpm8* expression in pro-inflammatory BMDMs, sustaining the hypothesis that this channel may be a druggable target in inflammation. Luteolin was able to revert icilin-mediated Ca^2+^ influx also in macrophages, further validating its ability to block Trpm8. Exposure to PAMPs (i.e., LPS and IFN-γ) triggers a rapid elevation in cytosolic Ca^2+^ that is mandatory for the pro-inflammatory switch of macrophages and the release of pro-inflammatory mediators [53]. Previous works showed that Orai channels, the main channels involved in store-operated Ca^2+^ entry, are not critical for LPS-induced macrophage activation [53]. More recently, the chanzyme TRPM7, another member of the TRPM subfamily of ion channels, was linked to the LPS-mediated Ca^2+^ influx in macrophages [36], suggesting that TRP channels may be instead implicated in such a mechanism. Here we demonstrate that LPS-induced Ca^2+^ elevations are severely blunted in *Trpm8*^-/-^ macrophages and, accordingly, TRPM8 blockade with luteolin also reduced cytosolic Ca^2+^ in response to LPS. Collectively, our data supports the prominent role of this ion channel in the macrophage response to pro-inflammatory stimuli. The question remains as to how TRPM8 is activated in macrophages. Recent studies suggest that several sensory TRP channels can be directly activated by LPS, but the concentrations required exceed those tested in this study [54–56]. Importantly, it has been shown that TRPM8 is constitutively activated in sensory neurons [57, 58], a phenomenon potentially mirrored in macrophages, based on our findings. Further research is needed to understand how TRPM8 mediates the intracellular Ca^2+^ increase triggered by LPS in macrophages, possibly through endogenous ligands or regulatory mechanisms like constitutive phosphorylation [59].

Irrespective of the mechanism underlying TRPM8 activation, we reveal that macrophages from *Trpm8*^*−/−*^ mice displayed blunted inflammatory responses upon challenging with pro-inflammatory stimuli. In accordance with these findings, TRPM8 modulation with luteolin induced a pro-tolerogenic profile in macrophages. Indeed, TRPM8 pharmacological inhibition increased the levels of IL-10 and suppressed the release of pro-inflammatory mediators IL-1β, IL-6, and TNF-α from BMDMs in vitro. IL-10 is a key immunosuppressive cytokine in activated macrophages, controlling essential metabolic pathways and reducing glucose uptake [40]. Specifically, exposure to PAMPs induces a metabolic shift in macrophages, promoting pro-inflammatory polarization marked by increased aerobic glycolysis and disrupted tricarboxylic acid cycle, leading to accumulation of succinate and citrate [41]. Our data show that TRPM8 pharmacological inhibition or genetic deletion reduced the glycolytic metabolism in pro-inflammatory macrophages, restoring a naïve-like metabolic profile. From the analysis of metabolites, luteolin-treated pro-inflammatory macrophages produced lower levels of succinate, but higher levels of lactate from early timepoints (i.e., 3, 6 h) compared to untreated cells. Lactate acts as a complex immunomodulatory molecule that controls innate and adaptive immune cell functions [47, 48]. In pro-inflammatory macrophages, lactate-derived histone lactylation serves as an epigenetic modulation that stimulates the expression of pro-tolerogenic genes, including IL-10 [47, 48]. We demonstrated that intracellular lactate in luteolin-treated BMDMs largely regulated IL-10 expression. In the absence of lactate, luteolin failed to induce IL-10 expression in pro-inflammatory macrophages, and, consistently, in the absence of IL-10 luteolin was not able to induce a pro-tolerogenic profile in LPS + IFN-γ stimulated macrophages.

Finally, the reconstitution of wild-type mice with *Trpm8*-deficient BM resulted in reduced severity of DSS-induced colitis. This chemically- induced model of acute colitis, is mostly dominated by an early innate immune response [60]. In line, in our data, Trpm8 was mostly expressed by myeloid cells during inflammation, but not by pro-resolving CD206^+^ macrophages. Accordingly, luteolin in vivo reduced colitis severity and mucosal injury caused by DSS administration. Both mice reconstituted with *Trpm8*-deficient BM, and WT mice orally treated with luteolin showed an impairment in the innate immune response during the recovery phase, thereby suppressing colitogenic responses. Overall, our data reveal TRPM8 as a major controller of pro-inflammatory response by myeloid cells in vivo.

## Conclusions

Our findings highlight the potential of targeting TRPM8 through dietary interventions to prevent chronic inflammatory diseases such as IBD. This research is of high significance as it pinpoints specific nutrients that could aid IBD patients, paving the way for crafting specialized diets and dietary supplements tailored for managing gastrointestinal inflammatory disease.

## Materials and methods

### Materials

Luteolin was purchased from ChemCruz (cat. sc-203119C, Santa-Cruz Biotechnology). GSK 2837808A and N-(3-aminopropyl)-2-[(3-methylphenyl) methoxy] -N-(2-thienylmethyl) benzamide hydrochloride (AMTB) were purchased from Tocris (cat. 5189 and 3989, Bio-Techne). For in vivo studies, luteolin was dissolved in 1% carboxymethyl cellulose (CMC), and for in vitro studies in 0.1% ethanol. GSK 2837808A was dissolved in 0.1% dimethyl sulfoxide (DMSO). AMTB was dissolved in mq-water. Vehicles did not affect the responses under the study. All the reagents for in vitro cell cultures and ex vivo analysis were provided by Merck Sigma (Merck Life Science), Aurogene, Thermo Fisher Scientific, Corning, and Bio-Rad.

### In silico studies

Molecular docking calculations were conducted through the Autodock Vina of PyRx 0.8 software, as previously described [61]. Crystal structures of target protein were derived from the Protein Data Bank (PDB) with PDB ID as follows: 6NR3 [transient receptor potential M8 (TRPM8)]. Discovery studio 2020 visualizer was employed to investigate the protein–ligand nonbonding interactions.

### Animals

Eight weeks old female wild-type C57BL/6 J (Envigo, RRID:IMSR_ENV:HSD-057), and *Trpm8*^*−/−*^ (B6.129P2-Trpm8tm1Jul/J, Stock No. 008198; Jackson Laboratories, RRID:IMSR_JAX:008198) mice were housed, at the Department of Pharmacy, University of Naples Federico II (Italy) animal facility. Trpm8^−/−^ mice have been previously described [29] and they develop normally without any spontaneous disease emerging under the breeding conditions [62]. Eight weeks old female wild-type C57BL/6 J (Envigo) and *IL-10*^*fl/fl*^ wild-type and LysMCre were kindly gifted from B. Stijlemans, Vrije Universiteit Brussel (VUB), and housed at KU Leuven animal facility (Belgium). All mice were housed under controlled conditions standard laboratory conditions (12-hr light/dark cycle, temperature of 22 ± 2 °C) and fed ad libitum with commercially available chow (ssniff® R/M-H, ssniff Spezialdiäten GmbH). Mice were randomly allocated to different experimental groups, and outcome assessments were performed single blind. All animals were humanely killed by carbon dioxide and cervical dislocation. Death was further assessed by confirmation of rigor mortis. All efforts were made to minimize the number of animals used and their suffering. All experimental procedures and protocols were in compliance with National (Direttiva 2010/63/UE) laws and policies and approved by the Italian Ministry of Health (Protocol Numbers 1101/2015-PR and 481/2020-PR) and by the Animal Care and Animal Experiments Ethical Committee of KU Leuven (188/2019 and P208/2018).

### Generation of mice bone marrow chimeras

6-week-old wild-type C57BL/6 J (Envigo, RRID:IMSR_ENV:HSD-057) mice were lethally irradiated with 9.5 Gy. Irradiation was performed on a LISA irradiator, XSTRAHL. Mice were reconstituted by intravenous administration of 10 × 10^6^ bone marrow (BM) cells from *Trpm8*^*−/−*^ mice (B6.129P2-Trpm8tm1Jul/J, Stock No. 008198; Jackson Laboratories, RRID:IMSR_JAX:008198). *Trpm8*^*-/-*^ chimera mice were left for at least 8 weeks before being subjected to DSS-induced colitis as described below.

### DSS-induced colitis

Colitis was induced in female wild-type C57BL/6 J or *Trpm8*^*-/-*^ chimera mice by DSS administration. DSS (molecular mass: 36 000–50 000 Da; MP Biomedicals, California, USA) was added to drinking water at 2.25% (w/v) for 5 days, followed by 10-day tap water period [60]. Mice were monitored daily for weight loss, stool consistency and haematochezia. Disease activity index was set as the combined score of weight loss (score=0: <1%, 1: ~1%–5%, 2: ~5%–10%, 3: ~10%–20%, 4: >20%), stool blood (score=0: absence, 2: presence, 4: gross bleeding) and stool consistency (score=0: formed and hard, 1: formed but soft, 2: loose stools, 3: mild diarrhea, 4: gross diarrhea), as previously reported [60]. Luteolin (3, 10, 30 mg/Kg) or vehicle (1% CMC) was administered by oral gavage every day for the entire duration of the experiments, starting one week before DSS administration. Mice were sacrificed at day 5, 10 and 15 after colitis induction, and the colon was resected for further investigation. No animal was removed from the study unless humane endpoints (e.g., body weight loss >20%, reduced food intake, abnormal posture) were reached.

### Isolation of epithelial and lamina propria cells

After removing fat and feces, colons were opened longitudinally, washed in Hank’s balanced salt solution (HBSS; cat. H9394, Sigma-Aldrich) supplemented with 1% fetal bovine serum (FBS), 100 μg/mL penicillin, and 100 μg/mL streptomycin, and cut into 0.5 cm pieces. The tissue was incubated twice at 37 °C with shaking for 15 min in HBSS containing 1 mM EDTA and 1% FBS. Strainer filtrates were centrifuged (5 min, 300 g) to collect epithelial cells. The remaining tissue sample was minced and incubated with prewarmed 1× Minimum Essential Medium (MEM)-α (Invitrogen) containing 2 mM L-glutamine, 100 μg/mL penicillin, 100 μg/mL streptomycin, 2- mercaptoethanol, and 10% FBS, and supplemented with 1.25 mg/mL collagenase D (cat. 11088882001, Sigma-Aldrich), 0.85 mg/mL collagenase V (cat. C9263-1G, Sigma-Aldrich), 1 mg dispase (cat. 17105-041, Gibco) and 30 U/mL DNase (cat. 04159001, Sigma-Aldrich) for 20 min in a shaking incubator at 37 °C. The resulting cell suspension was filtered through a 70 μm cell strainer and lamina propria cells were collected after centrifugation (5 min, 500 g).

### Flow cytometry

Single-cell suspensions (obtained as described above) were incubated for 15 min with mouse FcR Blocking Reagent (1:100 BD Pharmingen) at 4 °C. Next, cells were labeled with anti-CCR2-BUV395 (cat. 747972, BD Biosciences, clone: 475301, RRID: AB_2872433), Viability-eFluor 455 (cat. 65-0868-14, Invitrogen), anti-CD4-BUV496 (cat. 612952, BD Biosciences, clone: GK1.5, RRID: AB_2813886), anti-CD9-BUV563 (cat. 741270, BD Biosciences, clone: KMC8, RRID: AB_2870811), anti-CD11b-BUV737 (cat. 612801, BD Biosciences, clone: M1/70, RRID: AB_2738811), anti-CD11c-BV421 (cat. 117329, BioLegend, clone: N418, RRID: AB_11219593), anti-CD3-eFluor450 (cat. 48-0032-82, Invitrogen, clone: 17A2, RRID: AB_1272193), anti-CD19-BV605 (cat. 115539, BioLegend, clone: 6D5, RRID: AB_2563067), anti-Ly6C-BV650 (cat. 128049, BioLegend, clone: HK1.4, RRID: AB_2800630), anti-CD64-BV711 (cat. 139311, BioLegend, clone: X54-5/7.1, RRID: AB_2563846), anti-Ly6G-BV785 (cat. 127645, BioLegend, clone: 1A8, RRID: AB_2566317), anti-CD45-Spark Blue 550 (cat. 103165, BioLegend, clone: 30-F11, RRID: AB_2819791), anti-TRPM8-PE (cat. NBP1-97311PE, NovusBio), anti-CD206-PE Dazzle 594 (cat. 141732, BioLegend, clone: C068C2, RRID: AB_2565931), anti-NK1.1-PE-Cy5 (cat. 108716, BioLegend, clone: PK136, RRID: AB_493590), anti-MHC-II-AF700 (cat. 56-5321-82, Invitrogen, clone: M5/114.15.2, RRID: AB_494009), and anti-CD8a-APC-Cy7 (cat. 100714, BioLegend, clone: 53-6.7, RRID: AB_312752) for 20 min at 4 °C. Finally, cells were washed and resuspended in FACS buffer (0.5% FBS and 2 mM EDTA in PBS). Flow cytometry analyses were performed on a Sony ID7000 spectral analyzer (Sony) and subsequently analyzed using FlowJo v.10.8.1. T-cells (CD3^+^CD4^+/-^CD8^+/-^), NK cells (CD3^+/-^NK1.1^+^), B-cells (CD19^+^), DC (CD11c^+^), neutrophils (CD11b^+^Ly6G^+^), monocytes (CD11b^+^CD64^+^Ly6G^−^Ly6C^+^MHCII^−^), immature Mφ (CD11b^+^CD64^+^Ly6G^−^Ly6C^+^MHCII^+^) and mature Mφ [CD11b^+^ CD64^+^Ly6G^−^Ly6C^−^MHCII^+^ (CCR2^+^, CD206^+^)] cells were identified in the CD45^+^LD^-^ gate (Supplementary Fig. 7). Regarding the tSNE plots, the analysis was performed on a total of 45000 CD45^+^ cells. The cells were exported, concatenated, and analyzed with Tsne + FlowSOM Flowjo plugin (perplexity: 60, Max iterations: 1000). Following dimensional reduction, coordinates for each t-SNE dimension (i.e., tSNE1 and tSNE2) in the two-dimensional plots were determined and integrated as novel parameters.

### Histology

For histological analyses, the entire colons were taken, and tissues were flushed with phosphate-buffered saline (PBS), then swiss rolls were prepared as previously described [63]. Briefly, Swiss rolls were fixed in 4% neutral buffered formalin, processed for paraffin embedding and routine hematoxylin and eosin staining. Images were acquired using a Zeiss Axio Imager microscope and features were blindly scored for the presence of goblet cell loss, crypt density, hyperplasia, and submucosal infiltrate using the following grading system: Goblet cell loss 0 = none, 1 = < 10%, 2 = 10–50%, 3 = > 50%; Crypt density 0 = normal, 1 = decrease of <10%, 2 = decrease of ≥10%; Hyperplasia 0 = none, 1 = slightly increased crypt length, 2 = 2–3 times increased crypt length, 3 = > 3 times increased crypt length; Submucosal infiltrate 0 = none, 1 = individual cells, 2 = infiltrate[s], 3 = large infiltrate[s] [64].

### Immunofluorescence staining

Cultured WT BMDMs were fixed with methanol for 15 min at -20 °C, washed with DPBS, and blocked with normal donkey serum in TBS-T for 15 min at RT. Slides of colon tissue (5 μm) were deparaffinized with HistoChoice® Clearing Agent (cat. H2779, Sigma-Aldrich) and rehydrated with decreasing gradient alcohol. Antigen retrieval was performed using TrisEDTA at 95 °C for 20 min. Non-specific binding was blocked using blocking solution [1% Bovine Serum Albumine (BSA) in PBS 0.1% Triton™ X-100, 0.1% Tween20] for 1 h in a dark humid chamber. The slides were incubated in a humidified chamber overnight at 4 °C with with the following fresh primary antibodies: TRPM8 (1:100; cat. OSR00077W, Thermo Fisher, RRID:AB_2208883), IBA1 (1:250, Cat.# 234009, Clone Ch311H9, Synaptic Systems, RRID:AB_2891282), diluted in blocking solution. Therefore, secondary antibodies, Cy3 donkey anti-chicken (cat. 703-165-155 Jackson ImmunoResearch, RRID:AB_2340363) and Cy5 donkey anti-rabbit (cat. 711-175-152, Jackson ImmunoResearch, RRID:AB_2340607) diluted at 1:500 in blocking solution were added and detected. 4′,6-diamidino-2-phenylindole (DAPI) was used for nuclear staining. Samples were quenched and mounted with Vector® TrueVIEW® Autofluorescence Quenching Kit (cat. SP-8400, Vector Laboratories). The images were acquired and detected under 63× magnification, by using a Zeiss LSM 780 microscope.

### Bone marrow derived macrophages (BMDMs)

Bone marrow was obtained from C57BL/6 J wild-type (WT), *Trpm8*^*-/-*^ mice, IL-10^fl/fl^ WT and IL-10^fl/fl^ LysMCre mice femurs and tibias. Cells were incubated in macrophage culture medium [Dulbecco’s modified Eagle’s medium (DMEM) 4,5 mg/L glucose containing 100 U/mL of penicillin-streptomycin, 1% L-Glutamine and 10% (*v/v*) fetal bovine serum (FBS)] supplemented with 30% L929-conditioned media for 7 days. BMDMs were then reseeded and pre-treated with luteolin or vehicle (EtOH 0.1%) for 1 h and finally polarized toward pro-inflammatory phenotype with IFN-γ (50 ng/mL) plus LPS (20 ng/mL), except where otherwise stated. In the experiments involving the LDHa inhibitor GSK 2837808 A, macrophages were re-seeded in the presence or absence of the LDHa inhibitor (10μM) for 12 h before proceeding to the luteolin treatment and polarization towards a pro-inflammatory phenotype.

### Cell lines

RAW 264.7 macrophages, purchased from ATCC (cat. TIB-71, LGC Standards, RRID:CVCL_0493), were cultured in DMEM supplemented with 10% (*v/v*) FBS, 1 mmol·L^−1^ l-glutamine, 1 mmol·L^−1^ sodium pyruvate, 0.1 mmol·L^−1^ non-essential amino acids and 100 U·ml^−1^ antibiotics (penicillin and streptomycin) at 37 °C in 5% CO_2_. Human embryonic kidney 293 (HEK-293) cells were grown on 100-mm-diameter Petri dishes as monolayers in MEM (Life Technology) supplemented with non-essential amino acids, 10% FBS and 2 mM l-glutamine and were maintained under 5% CO_2_ at 37 °C. Cell viability was evaluated by trypan blue exclusion, and cell lines were confirmed to be Mycoplasma free (by using Mycoplasma PCR Detection Kit, Cat. G238, ABM). The medium was changed every 48 h in conformity with the manufacturer’s protocols. Cell viability and proliferation was assessed as described in supplementary materials.

### TRP calcium assays

Human embryonic kidney 293 (HEK-293) stably over-expressing recombinant rat TRPM8, rat TRPA1, and human TRPV1 were generated and subsequently cell clones were selected following published procedures [65]. The stable overexpression of the TRPM8, TRPA1 and TRPV1 genes was periodically checked by quantitative real-time PCR following published procedures [66]. To determine the effect of luteolin and rutin, control blank (non-transfected) and transfected HEK293 cells were loaded with the methyl ester Fluo4-AM (cat. F14201, Thermo Fisher Scientific) 4 μM in DMSO containing 0.02% Pluronic F-127 (cat. P3000MP, Thermo Fisher Scientific) for 1 h in the dark at room temperature in MEM not containing FBS. After 1 h, cells were washed twice in Tyrode’s buffer (145 mM NaCl, 2.5 mM KCl, 1.5 mM CaCl_2_, 1.2 mM MgCl_2_, 10 mM D-glucose, and 10 mM HEPES, pH 7.4), re-suspended in Tyrode’s buffer, and transferred into the quartz cuvette of the spectrofluorimeter (Perkin-Elmer LS50B; PerkinElmer Life and Analytical Sciences) under continuous stirring. Change of the intracellular calcium concentration [Ca^2+^]i was determined in the presence of crescent concentrations of luteolin or rutin by measuring cell fluorescence at 25 °C (*λ*_EX_ = 488 nm, *λ*_EM_ = 516 nm). Curve fitting (sigmoidal concentration-response variable slope) and parameter estimation were performed with GraphPad Prism®8 (GraphPad Software Inc.). Potency was expressed as the concentration of test substances exerting a half-maximal agonist effect (i.e., half-maximal increases in [Ca^2+^]i (EC50) calculated by using GraphPad®. The desensitization (reported as antagonism) of the TRPA1, TRPV1, and TRPM8 channels was calculated by measuring the effect of luteolin, preincubated in the cells for 5 minutes before stimulation with AITC (100 μM, TRPA1 agonist), capsaicin (0.1 μM, TRPV1 agonist), or icilin (0.25 μM, TRPM8 agonist). IC50 is defined as the concentration of luteolin that induces half-maximal inhibition of the agonist response. The efficacy of the agonists was determined by normalizing its effect to the maximum Ca^2+^ influx effect on [Ca^2+^]i observed with the application of 4 μM ionomycin (cat. 11932, Cayman). Measurement of [Ca^2+^]i in TRPM8 cells was performed at 22 °C with a Fluorescence Peltier System (PTP-1, Perkin-Elmer).

### BMDM intracellular calcium assay

BMDMs were loaded with the methyl ester Fluo4-AM (cat. F14201) 1 μM, containing 0.01% Cremophor for 20 min in the dark at room temperature. After loading, the cells were rinsed with oxygenated Krebs solution and transferred to a recording chamber mounted on an inverted Zeiss Axiovert 200 M microscope (Zeiss). A gravity-fed perfusion system ensured continuous and constant perfusion (1 ml/min) of the preparation with Krebs solution (at room temperature) and excess solution was removed via a peristaltic suction pump. Fluo-4 was excited at 470 nm (exposure time: 100 ms), and its fluorescence emission was collected at 525/50 nm. Images were acquired at 2 Hz using TILLVision software (TILL Photonics). Change of the intracellular calcium concentration [Ca^2+^]_i_ was determined in presence of icilin (40 μM) for 60 s, LPS (100 ng/mL) for 30 s, or adenosine-5’-triphosphate (ATP, 10 μM) for 10 s, using a gravity-fed perfusion system. In order to induce pharmacological blocking of TRPM8, luteolin (10 μM) or AMTB (5 μM) were applied on the tissue surface via the gravity-fed perfusion system for 10 min, before icilin or LPS administration. Image analysis was performed using ImageJ. Fluorescence intensity was normalized to the basal fluorescence at the onset of the recording for each region of interest (*F*/*F*0), and area under the curve of each fluorescence peak was analyzed.

### Nitrite measurement by GRIESS assay

RAW 264.7 cells and BMDMs (WT and *Trpm8*^*−/−*^) were seeded in 24-well plates and treated with scalar non-toxic concentrations of luteolin (0.1–10 μM), stimulated or not with LPS (1 μg/mL) or IFN-γ (50 ng/mL) plus LPS (20 ng/mL), respectively. After 18 h incubation, supernatant was collected and The NO^2−^ concentration was measured with the Griess reagent in which naphthylethylenediamine dihydrochloride and sulfanilamide reacted with NO^2−^ to form a purple azo-product. The concentration of the azo-product was measured colorimetrically at 540 nm using a microplate reader. The experiments were performed as three independent biological experiments, each with three wells per sample for incubation (24-well plate) that was split to other three wells per sample for technical replicate measurements (96-well plate).

### Enzyme-linked immunosorbent assay (ELISA)

IL-6, IL-1β, TNF-α, and IL-10 levels were quantified in supernatant obtained from WT, *Trpm8*^*−/−*^, IL-10^fl/fl^ WT, and IL-10^fl/fl^ LysMCre BMDM cultures, using commercial ELISA kits (Invitrogen), according to the manufacturer’s instructions. Results are expressed as picograms per milliliter of protein extract. The experiments were performed as three to four independent biological experiments, each measured in a technical duplicate.

### Seahorse extracellular flux assay

For extracellular flux assay, RAW 264.7 and BMDMs (WT, *Trpm8*^*−/−*^, IL-10^fl/fl^ WT and IL-10^fl/fl^ LysMCre) were treated with luteolin (10 μM) and then stimulated with LPS (1 μg/mL) or IFN-γ (50 ng/mL) plus LPS (20 ng/mL) respectively, for 18 h. ECAR, basal and compensatory glycolysis, glycolytic capacity and non-glycolytic acidification were measured using XF24 Seahorse Extracellular Flux Analyzer following the manufacturer’s instruction.

### NMR based metabolomics

RAW 264.7 macrophages were grown on 100-mm-diameter Petri dishes as described in supplementary materials. Upon achievement of 80% cellular confluency, the culture medium was removed, and the cells were processed for the endo-metabolomic analysis. In brief, the cells were extensively washed (four times) with ice-cold phosphate-buffered saline (PBS 1X) in order to completely remove any residue of culture medium. Then, the cell plates were immersed into liquid nitrogen upon complete freezing of the samples and then slowly thawed in an ice bath. Afterwards, 5.4 mL of PBS was added to each culture dish and cells were collected by scraping with a rubber policeman. Finally, the cells were counted, placed in Falcon tubes and the final PBS volumes were adjusted to obtain 15 × 10^6 cells into 5.4 mL PBS (pH 7.4). Subsequently, the quenched cells were lysed by three short-pulse cycles of sonication of 30 s each, at maximum power, thus favoring the release of the intracellular metabolites. A dual-phase extraction procedure, introduced by Bligh and Dyer [67] with slight modifications, was employed to separate the polar metabolites from the non-polar compounds, as already reported elsewhere [68]. Briefly, 6 mL of cold methanol (−20 °C) and 6 mL of chloroform were added to the original aqueous solution (5.4 mL) containing quenched cells (volume ratio of 1:1:0.9). Then, the mixture was incubated for 20 min on ice, repeatedly vortexed to facilitate the extraction and centrifuged at 4000 *g* at 4 °C for 20 min, thus obtaining a two-phase extract. The skin-like layer between the two phases entrapped proteins and macromolecules. Subsequently, the upper phase, containing the water-soluble intracellular metabolites, was separated from the organic lower phase and carefully transferred into different falcon tubes. Solvents were completely removed from both fractions using a vacuum concentrator (hydrophylic phase) and under a gentle flow of N_2_ gas (organic phase). In this study, only the hydrophilic phase was considered, however the organic phase, rich in non-polar metabolites such as lipids, has been stored at −80 °C for future analysis. The aqueous cell extracts were dissolved in 700 μL of D_2_O, vortexed briefly, and transferred into 5-mm NMR tubes for the NMR analysis. Detailed descriptions of the 1H-NMR spectra acquisition parameters, metabolites identification procedure, NMR data pre-processing, and PCA are provided in the supplementary materials.

### Metabolite extraction and liquid chromatography-mass spectrometry analysis

Metabolites were extracted from BMDMs cell pellet by adding 800 μl 60% methanol (cat. A456-500, Fisher Scientific) containing 6.67 μg/ml glutaric acid (cat. G3407, Sigma-Aldrich) in LC/MS grade water (cat. W6500, Fisher Scientific). After the addition of 500 μl chloroform (cat. C2432, Sigma-Aldrich,), samples were vortexed for 10 min at 4 °C and then spun at 17,000 × *g* for 10 min at 4 °C. The upper phase was transferred into a fresh tube and dried down in a Vacufuge plus speed-vac at 4 °C. The metabolite extract was separated using an iHILIC-(P) Classic column (2.1 μm, 150 mm × 2.0 mm I.D., The Nest Group) coupled to a Thermo Scientific SII UPLC system. The autosampler and column oven were held at 4 °C and 25 °C, respectively. The iHILIC-(P) Classic column was used with buffer A (0.1% ammonium hydroxide, 20 mM ammonium carbonate) and buffer B (100% acetonitrile). The chromatographic gradient was run at a flow rate of 0.150 ml/min as follows: 0–20 min: linear gradient from 80% to 20% B; 20–20.5 min: linear gradient from 20% to 80% B; 20.5–28 min: hold at 80% B. The mass spectrometer was operated in full scan, negative ion model. Mass spectrometry detection was carried out on a Q Extractive HF-X orbitrap mass spectrometer with a HESI source. For metabolite quantification, TraceFinder software (ThermoFisher) was used. The lower phase after chloroform extraction was also dried down in a Vacufuge plus speed-vac at 4 °C and proteins were resuspended in 0.2 M NaOH and heated at 90 °C for 15 min. The protein concentration was determined using the Pierce BCA Protein Assay Kit Assay (cat. 23227, ThermoFisher). The metabolite levels were normalized by the total protein amount per sample in μg.

### Quantitative real-time polymerase chain reaction (qPCR)

The RNA extraction was performed using innuPREP RNA Mini Kit 2.0 (Analytik Jena) following the manufacturer’s instructions. Total RNA was retro-transcribed into complementary cDNA by qScript cDNA SuperMix (Quanta Biosciences) according to the manufacturer’s instructions. Quantitative real-time transcription PCR was performed with the LightCycler 480 SYBR Green I Master (Roche Applied Science) using the Light Cycler 480 (Roche). Results were quantified using the 2^−ΔCT^ method. The expression levels of the genes of interest were normalized to the expression levels of the reference gene RPLP32. Polymerase chain reaction experiments were performed in 3–5 replicates. The primer sequences used are listed in Table 1 and refer to murine species. The primers used in Supplementary Fig. 6 were purchased from Life Technologies [Assays-on-Demand Gene Expression Products, Mm00478374_m1 for cyclooxygenase-2 gene, Mm00443258_m1 for tumor necrosis factor (TNF)-α gene, Mm00440502_m1 for inducible nitric oxide synthase (iNOS) gene, Mm00446190_m1 for interleukin (IL)-6, Mm00434228_m1 for IL-1β, Mm01288386_m1 for IL-10 gene, Mm02619580_g1 for β-actin gene, Mm00446190_m1 for HIF1a gene].

**Table 1:** Primer sequences.

*Gene*	Forward Primer	Reverse Primer
***Arg1***	CAGAAGAATGGAAGAGTCAG	CAGATATGCAGGGAGTCACC
***Il10***	CCAAGCCTTATCGGAAATGA	TCACTCTTCACCTGCTCCAC
***Il1β***	GACCTTCCAGGATGAGGACA	TCCATTGAGGTGGAGAGCTT
***Il6***	CCATAGCTCCTGGAGTACAT	TGGAAATTGGGGTAGGAAGGA
***Mrc1***	GTTCACCTGGAGTGATGGTTCTC	AGGACATGCCAGGGTCACCTTT
***Rplp32***	AAGCGAAACTGGCGGAAAC	TAACCGATGTTGGGCATCAG
***Tnfα***	TCTTCTCATTCCTGCTTGTGG	CACTTGGTGGTTTGCTACGA

### DNA methylation assay

To analyze changes in the methylation status of the murine *Trpm8* in our experimental conditions, we performed a bioinformatic analysis using the free available software MethPrimer 2.0 for the identification of CpG islands and sites in the 5’-untranslated region (5’-UTR) and promoter regions (~2000 bp) of the gene. DNA was extracted from macrophages with the use of DNeasy Blood & Tissue Kit (Cat. No. 69504, Quiagen). Subsequently, 0.5 μg of DNA from each sample was treated with bisulfite, using the EpiTect Bisulfite Kit (Cat. No. 59104, Quiagen). The primers used to analyze the methylation/methylation status of *Trpm8* are reported in Table 2. Quantitative PCR conditions and validation were performed following published procedures [69]. The expression of the housekeeping gene ribosomal protein S16 on non-bisulfite converted DNA was used to normalize the Ct values.

**Table 2:** Primer sequences for *Trpm8* chromatin.

Primer	Sequence
**methylated forward 1 (MF1)**	ATGTTTAGAGGATTAGTGAATACGT
**methylated reverse 1 (MR1)**	AACTAAAAACACCATAAAAATCGTA
**unmethylated forward 1 (UF1)**	ATGTTTAGAGGATTAGTGAATATGT
**unmethylated reverse 1 (UR1)**	AAACTAAAAACACCATAAAAATCATA
**methylated forward 2 (MF2)**	ATGTTTAGAGGATTAGTGAATACGT
**methylated reverse 2 (MR2)**	AACTAAAAACACCATAAAAATCGTA
**unmethylated forward 2 (UF2)**	ATGTTTAGAGGATTAGTGAATATGT
**unmethylated reverse 2 (UR2)**	AACTAAAAACACCATAAAAATCATA
**methylated forward 3 (MF3)**	ATGTTTAGAGGATTAGTGAATACGT
**methylated reverse 3 (MR3)**	AACTAAAAACACCATAAAAATCGTA
**unmethylated forward 3 (UF3)**	ATGTTTAGAGGATTAGTGAATATGT
**unmethylated reverse 3 (UR3)**	ACTAAAAACACCATAAAAATCATA

### Statistical analysis

Results are shown as mean ± standard error of the mean (SEM). Significance between two mean groups was determined by unpaired or paired two-tailed Student’s *t* test, while one or two-way analysis of variance (ANOVA) followed by Dunnett’s or Tuckey’s multiple comparison test was performed to compare the mean of multiple groups. Outliers (if any) were identified by ROUT test. GraphPad Prism V.9.5.1 software (GraphPad Inc.) was used to generate graphs and to perform statistical analysis.

## Supplementary information

Supplementary Materials

## Data Availability

The authors declare that all the data supporting the findings of this study are available within the paper and its supplementary information files. No datasets were generated or analyzed during the current study.
