# Supplementary material for: Dietary targeting of TRPM8 rewires macrophage immunometabolism reducing colitis severity
Source: Cell Death Dis. 2025 Apr 25;16(1):343. doi: 10.1038/s41419-025-07553-9 (PMC12032354; doi:10.1038/s41419-025-07553-9)
Supplement: Supplementary file 1 — Supplementary Materials [file 41419_2025_7553_MOESM1_ESM.pdf]

Dietary targeting of TRPM8 rewires macrophage immunometabolism reducing colitis severity

Supplementary materials and methods

Ex vivo studies in colon tissues

Isolated colon specimens from WT C57BL/6J mice were maintained in a humidified incubator with 5% CO_2 at 37 ◦C for 4 h (incubation period), in RPMI buffer with added bacterial LPS (10 µg/mL), as previously described [1]. During the incubation period, the tissues were challenged with scalar concentrations of luteolin (0.1-30 ng/mL) for 18 h. Tissues were then processed for RT-qPCR analysis.

Cell viability assay

RAW 264.7 cells, WT and *Trpm8*$^{-/-}$ BMDMs were seeded in 96-well culture plates and treated for 24h with scalar concentrations of luteolin (0.1–300 µM), in the presence or not of IFN-γ (50 ng/mL) plus LPS (20 ng/mL). The plates were then incubated for 3h with a medium containing neutral red. The cells were subsequently washed, the dye extracted in each well with 1% acetic acid and the absorbance read using a microplate spectrophotometer at 540 nm. Viability was expressed as % of control group. The experiments were performed as three independent biological experiments, each with 5 wells per sample for technical replicates.

Cell proliferation assay

WT BMDMs were seeded in 96-well flat-bottom culture plates and treated with scalar concentrations of luteolin (0.1–80 µM), in the presence or not of IFN-γ (50 ng/mL) plus LPS (20 ng/mL). The plates were then placed into the IncuCyte® ZOOM System (Sartorius) and cell growth was assessed for 48 hours. The software was adjusted to take 2 images per well every 2 hours. Analysis was carried out according to manufacturer's instructions.

NMR based metabolomics

All one-dimensional 1H-NMR spectra were acquired at 300 K on a Bruker Avance NEO 600 MHz spectrometer (Bruker Biospin Gmbh, Rheinstetten, Germany) equipped with a QCI cryo-probe set for 5 mm sample tubes and a cooled autosampler (SampleJet, at 300 K). The 1H NMR spectra of hydrophilic cell extracts were acquired with Topspin 4.1 (Bruker Biospin GmbH, Rheinstetten, Germany), using the 'noesygppr1d' pulse sequence allowing for a quantitative evaluation even close to the water signal [2] which was presatured at 4.698 ppm. All the experiments were acquired with an acquisition time of 3.67 s, a relaxation delay of 4 s, receiver gain of 101, 128 scans, 4 dummy scans and a spectral width of 17,857 Hz (29.755 ppm). All samples were automatically tuned, matched and shimmed. Prior to Fourier transformation, the free induction decays were multiplied by an exponential function equivalent to a 0.3-Hz line-broadening factor. Then, the transformed spectra were

automatically corrected for phase and baseline distortions and calibrated using TopSpin built-in processing tools.

The assignment of the hydrophilic metabolites was achieved by (i) analysis of literature data [3], [4], [5]; (ii) comparison with the chemical shifts of the metabolites in the Human Metabolome Database (HMDB); (iii) peak fitting routine within the spectral database in Chenomx NMR Suite 8.8 software package (Chenomx, AB, Canada) in its evaluation version.

NMR spectra were then imported into MATLAB (R2015b; Mathworks, Natick, MA) where the spectral regions above 10 ppm and below 0 ppm were removed because they contained only noise. To correct for spectral misalignment, an interval-based alignment step was carried out using the icoshift algorithm [6]. Then, in order to reduce the model complexity, the peak areas of the well-separated and safely assigned resonances of 13 selected metabolites were manually integrated and submitted to the data analysis as a data matrix made of 6 rows (samples) x 13 columns (metabolites). Such data matrix was then submitted to the PLS toolbox version 8.6.1 (Eigenvector Research, Manson, USA) under MATLAB environment, version R2015b (MathWorks Inc., Massachusetts, USA) to perform Principal Components Analysis (PCA). Prior to the analysis, data was normalized, according to the total area (1-norm) and then autoscaled. Autoscaling employs both the standard deviation as a scaling factor thus giving all metabolites the same chance to affect the model and the mean-centering, which is needed to compute PCA.

PCA is an unsupervised pattern recognition method that allows the reduction of the dimensionality of a data set consisting of a large number of interrelated variables, providing a visual representation of the major variance in the data [7]. Thus, the original variables are transformed into a smaller set of new uncorrelated variables, called principal components (PCs), which are ordered according to the explained variance that they are able to retain. Most of the relevant systematic information is usually calculated by the first few PCs, while the following ones are often computed considering chance variation and noise. The outcome of this analysis consists of two plots: a scores plot, where the samples are displayed as scores, and a loadings plot that shows which are the variables responsible for the distribution of the samples observed in the scores plot. Thus, samples having similar scores will cluster together as well as variables with comparable loadings will be close, meaning that they are highly correlated.

References

[1] L. Recinella *et al.*, 'A grape (Vitis vinifera L.) pomace water extract modulates inflammatory and immune response in SW-480 cells and isolated mouse colon', *Phytotherapy Research*, vol. 36, no. 12, 2022, doi: 10.1002/ptr.7581.

[2] N. Iaccarino *et al.*, 'Ancient danish apple cultivars—A comprehensive metabolite and sensory profiling of apple juices', *Metabolites*, vol. 9, no. 7, 2019, doi: 10.3390/metabo9070139.

[3] I. Lauri *et al.*, 'Development of an optimized protocol for NMR metabolomics studies of human colon cancer cell lines and first insight from testing of the protocol using DNA G-quadruplex ligands as novel anti-cancer drugs', *Metabolites*, vol. 6, no. 1, 2016, doi: 10.3390/metabo6010004.

[4] A. L. Fuchs *et al.*, 'Quantitative 1H NMR metabolomics reveal distinct metabolic adaptations in human macrophages following differential activation', *Metabolites*, vol. 9, no. 11, 2019, doi: 10.3390/metabo9110248.

[5] F. Vrieling *et al.*, 'Analyzing the impact of Mycobacterium tuberculosis infection on primary human macrophages by combined exploratory and targeted metabolomics', *Sci Rep*, vol. 10, no. 1, 2020, doi: 10.1038/s41598-020-62911-1.

[6] F. Savorani, G. Tomasi, and S. B. Engelsen, 'icoshift: A versatile tool for the rapid alignment of 1D NMR spectra', *Journal of Magnetic Resonance*, vol. 202, no. 2, 2010, doi: 10.1016/j.jmr.2009.11.012.

[7] H. Hotelling, 'Analysis of a complex of statistical variables into principal components.', *J Educ Psychol*, vol. 24, no. 6, pp. 417–441, Sep. 1933, doi: 10.1037/h0071325.

Supplementary Figures and Tables

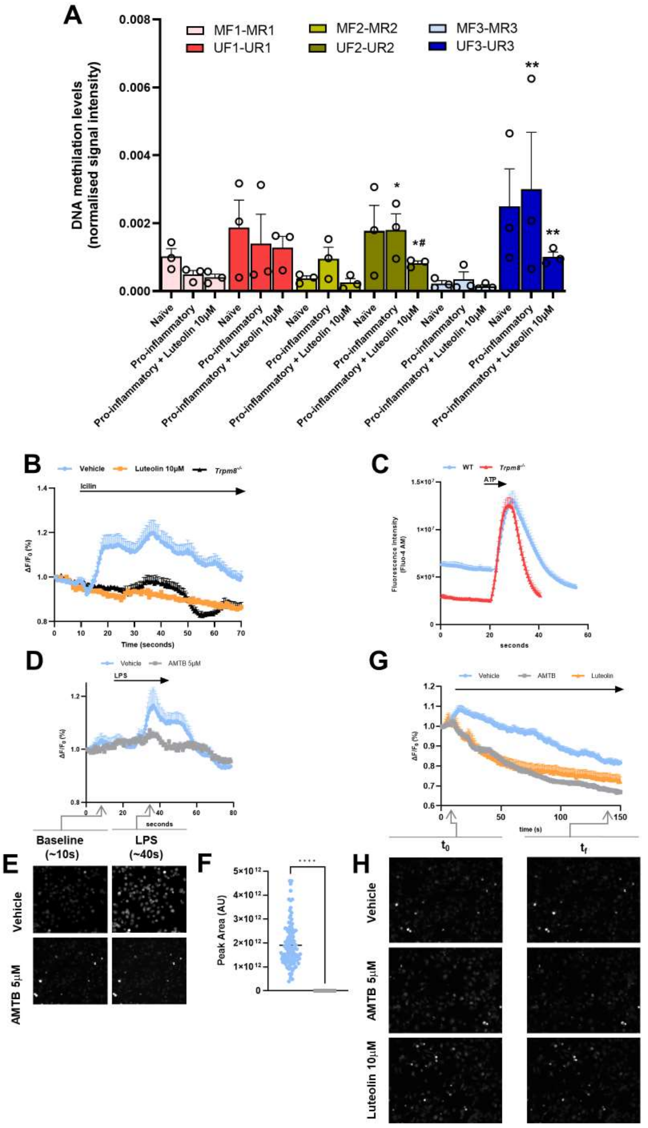

Supplementary Figure 1

Supplementary Figure 1. (A) *Trpm8* DNA methylated vs unmethylated expression level detected by qPCR in naive and pro-inflammatory BMDMs treated or not with luteolin (10 μM). Data are presented as normalised Relative Fluorescence Units (RFU) to the housekeeping gene S16 (input control) of n=3 independent experiments. Error bars represent ± SEM. P value was determined using unpaired Student's t-test. *p<0,05; **p<0,01 *vs* MF-MR; # p<0,05 *vs* Pro-inflammatory UF2-UR2. (B) Relative changes in the fluorescence of Fluo-4AM-loaded WT and *Trpm8*$^{-/-}$ BMDMs, reflecting changes in intracellular $^{Ca2+}$ concentration [Ca^{2+}]i after icilin (40 μM) perfusion for 1 min, in the presence or not of luteolin (10 min pre-incubation). Data are presented as the average of n=3 independent experiments. Error bars represent ± SEM. (C) Relative changes in the fluorescence of Fluo-4AM-loaded WT and *Trpm8*$^{-/-}$ BMDMs, reflecting changes in intracellular Ca^{2+} concentration [Ca^{2+}]i after ATP (10 μM)

perfusion for 10 s. Data are presented as the average of n=3 independent experiments. Error bars represent ± SEM. (D) Relative changes in the fluorescence of Fluo-4AM-loaded BMDMs, reflecting changes in intracellular Ca^{2+} concentration $[Ca^{2+}]i$ BMDMs after LPS (100 ng/mL) perfusion for 30 s, in the presence or not of AMTB (10 min pre-incubation). Data are presented as the average of n=3 independent experiments. Error bars represent ± SEM (n = 108 and n=144 for Vehicle and AMTB 5 μM, respectively). (E) Representative fluorescence images from indicated time points in the experiments shown in panel D. (F) Statistical representation of total peak area of Fluo-4 fluorescence after LPS perfusion from panel D. Error bars represent ± SEM. P value was determined using unpaired Student's t-test. ****p<0,0001. (G) Relative changes in the fluorescence of Fluo-4AM-loaded BMDMs, reflecting changes in intracellular Ca^{2+} concentration $[Ca^{2+}]i$ BMDMs after vehicle (Krebs solution), AMTB (5 μM) or luteolin (10 μM) perfusion for ~150 s. Data are presented as the average of n=3 independent experiments. Error bars represent ± SEM (n=144 for all the experimental groups). (H) Representative fluorescence images from indicated time points in the experiments shown in panel G.

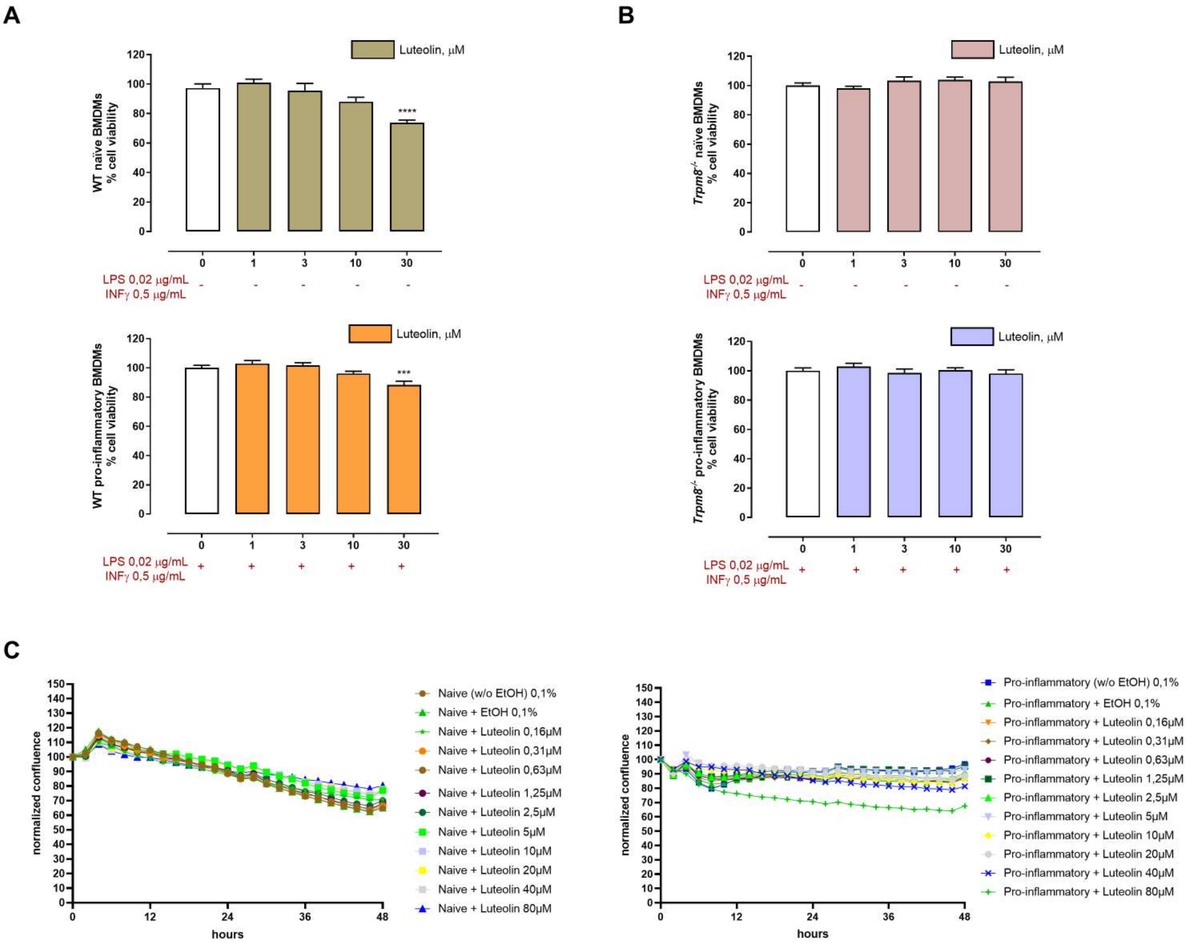

Supplementary Figure 2

Supplementary Figure 2. (A-B) Cell viability percentage of naïve and pro-inflammatory WT (A) or *Trpm8*$^{-/-}$ (B) BMDMs alone or in the presence of luteolin (1–30 μM) after 18 h. Data are presented as the average of n=3 independent experiments. Error bars represent ± SEM. P value was determined using one-way ANOVA followed by Tukey's multiple comparisons test. ***p<0,001; ****p<0,0001. (C) Cell viability rate of naïve and pro-inflammatory WT BMDMs alone or in the presence of luteolin (0,16–80 μM) up to 48 h. Data are presented as the average of n=3 independent experiments. Error bars represent ± SEM.

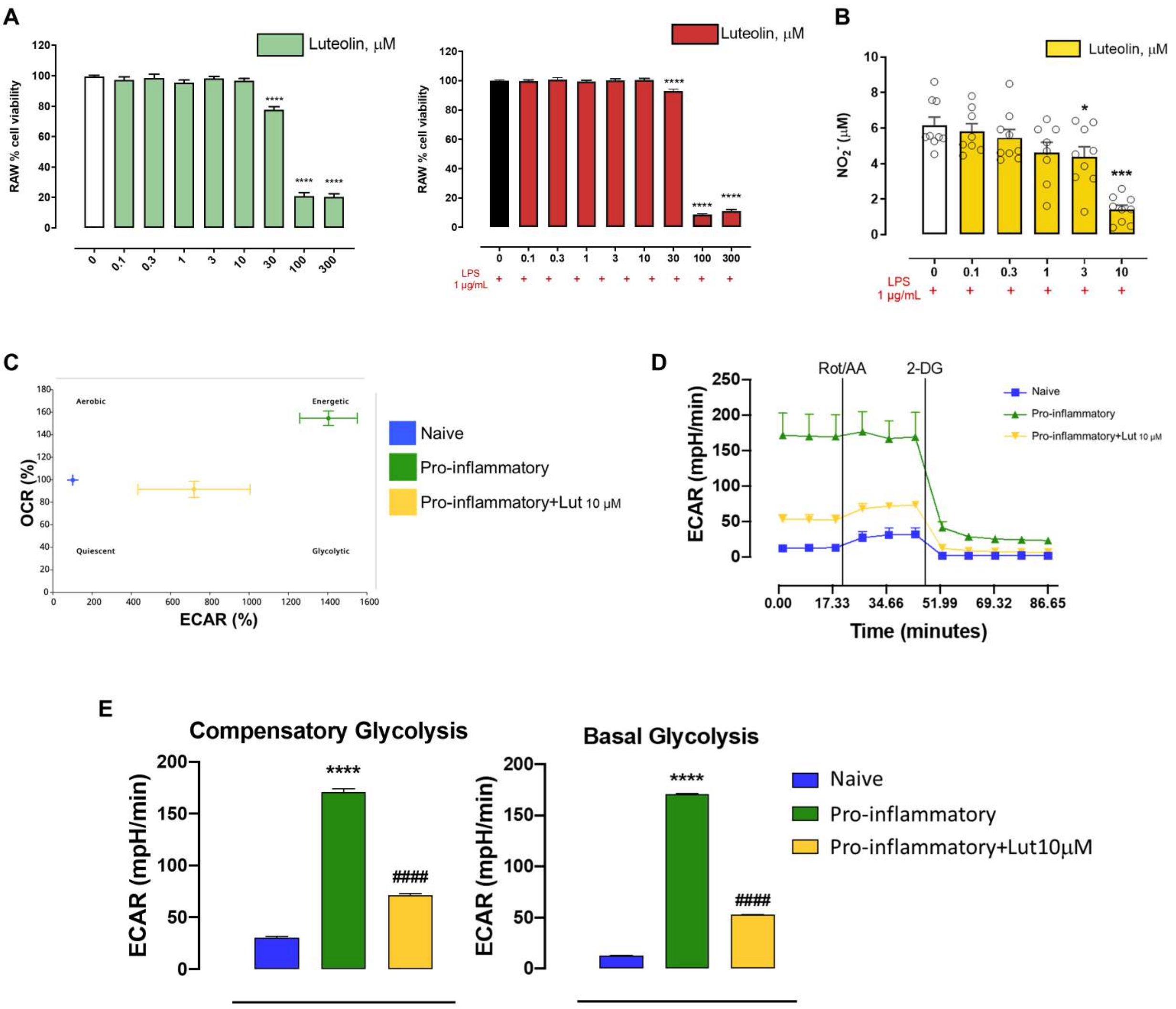

Supplementary Figure 3

Supplementary Figure 3. (A) Cell viability percentage of naïve and LPS-stimulated RAW 264.7 alone or in the presence of luteolin (0,1–300 μM) after 18 h. Data are presented as the average of n=3 independent experiments. Error bars represent ± SEM. P value was determined using one-way ANOVA followed by Tukey's multiple comparisons test. ****p<0,0001. (B) Nitrite levels measured in the supernatant of LPS-stimulated RAW 264.7 alone or in the presence of luteolin (1–10 μM) for 18 h. Data are presented as the average of n≥3 independent experiments, with each dot representing an individual cell passage. Error bars represent mean ± SEM. P value was determined using one-way ANOVA followed by Tukey's multiple comparisons test. *p<0,05; ***p<0,001. (C-D) Energetic map (C), and ECAR kinetics (D) of naïve (M0) and LPS-stimulated (M1) RAW 264.7 treated or not with Luteolin (10 μM). The energetic map was calculated on the basis of OCR and ECAR values measured by Seahorse analysis. Data are presented as the average of n=5 independent experiments. Error bars

represent ± SEM. (E) Glycolytic parameters of naïve and LPS-stimulated (pro-inflammatory) RAW 264.7 treated or not with Luteolin (10 μM). Data are presented as the average of n=5 independent experiments. Error bars represent ± SEM. P value was determined using one-way ANOVA followed by Tukey's multiple comparisons test. ****p<0,0001 *vs* naive; #### p<0,0001 *vs* Pro-inflammatory.

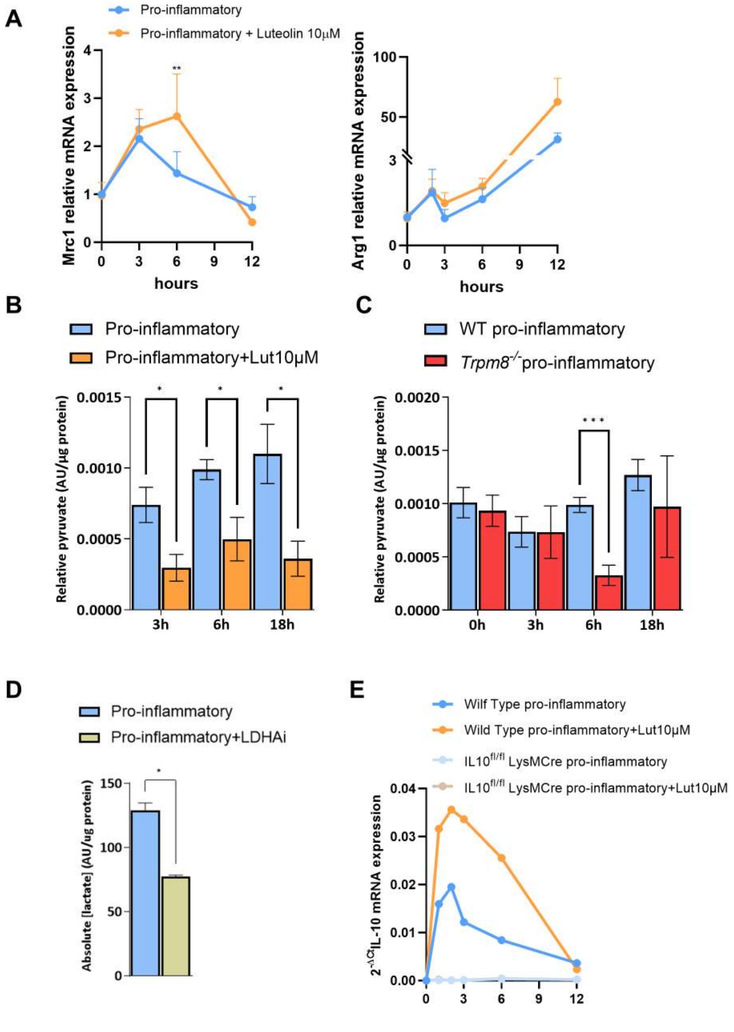

Supplementary Figure 4

Supplementary Figure 4. (A) *Mrc1* and *Arg1* mRNA measured in pro-inflammatory WT BMDMs treated or not with luteolin (10 µM). The analysis was performed at baseline (0 h) and after 1, 3, 6 or 12 h of polarization with IFN-γ plus LPS. Data are presented as the average of n≥3 independent experiments. Error bars represent ± SEM. P value was determined using ratio-paired Student's t-test. *p<0,05. (B) Relative pyruvate levels measured by GC-MS metabolomic analysis of pro-inflammatory WT BMDMs treated or not with luteolin (10 µM). The analysis was performed after 3, 6 or 18 h of polarization with IFN-γ plus LPS. Data are presented as peak area normalized on µg of protein of n=3 independent experiments, each performed in technical triplicate. Error bars represent ± SEM. P value was determined using unpaired Student's t-test. *p<0,05. (C) Relative pyruvate levels measured by GC-MS metabolomic analysis of pro-inflammatory WT or *Trpm8*$^{-/-}$ BMDMs. The analysis was performed in basal conditions (0h) and after 3, 6 or 18h of polarization with IFN-γ plus LPS. Data are presented as peak area normalized on µg of protein of n=3 independent experiments, each performed

in technical triplicate. Error bars represent ± SEM. P value was determined using unpaired Student's t-test. ***$p<0,001$. (D) Absolute lactate levels measured by GC-MS metabolomic analysis of pro-inflammatory WT BMDMs treated or not with LDH-a inhibitor GSK2837808A (10 μM). The analysis was performed after 6 h of polarization with IFN-γ plus LPS. Data are presented as peak area normalized on μg of protein of n=3 independent experiments. Error bars represent ± SEM. P value was determined using unpaired Student's t-test. *$p<0,05$. (E) IL-10 mRNA measured on *IL-10*$^{fl/fl}$ WT and *IL-10*$^{fl/fl}$ LysMCre BMDMs treated or not with luteolin (10 μM). The analysis was performed after 0, 1, 3, 6 or 12 h of polarization with IFN-γ plus LPS. Data are presented as the average of n=5 independent experiments.

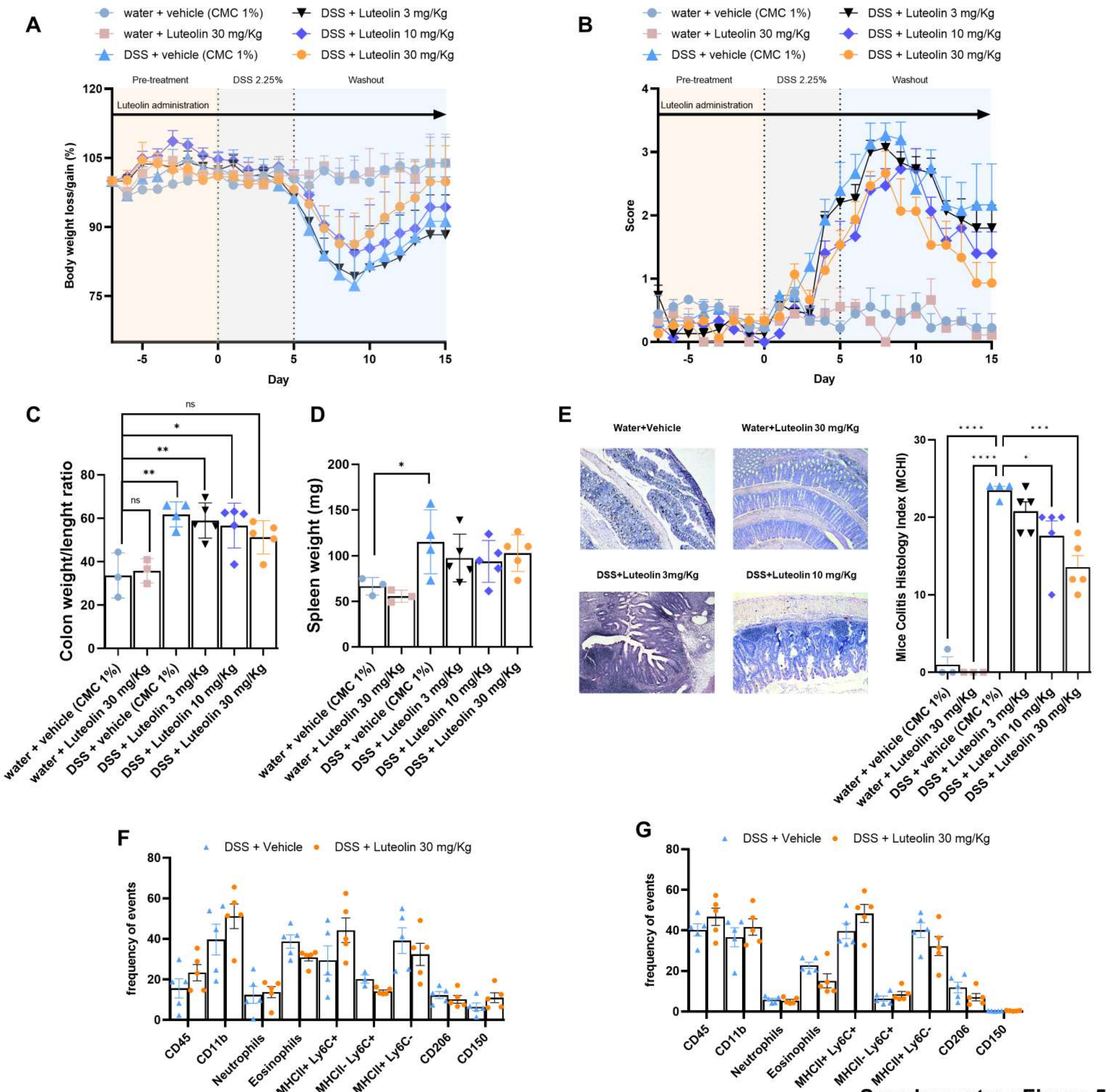

Supplementary Figure 5

Supplementary Figure 5. (A) Mice body weight gain/loss. Data are presented as the average of n=10 mice for each experimental group. Error bars represent ± SEM. (B) Disease activity index (DAI) of colitis severity. Data are presented as the average of n≥10 mice for each experimental group. Error bars represent ± SEM. (C-D) Colon weight/length ratio (C) and spleen weight (D) collected 15 dpt. Data are presented as the average of n=10 mice for each experimental group. Error bars represent ± SEM. P value was determined using one-way ANOVA followed by Tukey's multiple comparisons test. ns= non significant; *p<0,05; **p<0,01. (E) Histological score (dx) and representative H&E images (sx) of mice colon sections. Error bars represent mean ± SEM. P value was determined using one-way ANOVA followed by Dunnett's multiple comparisons test. *p<0,05; ***p<0,001; ****p<0,0001. (F-G) Percentage of CD45, CD11b, Ly6g neutrophils, Ly6c and MHCII, CCR2 and CD206 populations in lamina propria of mice colon 5 (F) and 10 (G) dpt. Data are presented as the average of n=5 mice for each experimental group. Error bars represent ± SEM.

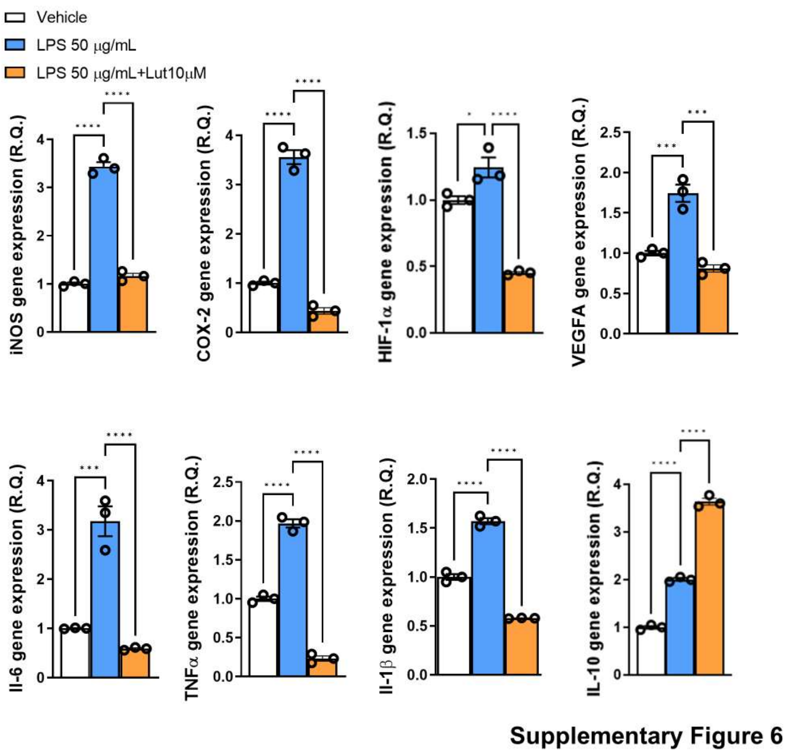

Supplementary Figure 6. iNOS, COX-2, HIF1-α, VEGFA, IL-6, TNF-α, IL-1β and IL-10 relative mRNA levels measured on LPS-stimulated WT mice colon treated or not with Luteolin (10 μM). Data are presented as the average of n=3 independent experiments. Error bars represent ± SEM. P value was determined using one-way ANOVA followed by Tukey's multiple comparisons test. *p<0,05; ***p<0,001; ****p<0,0001.

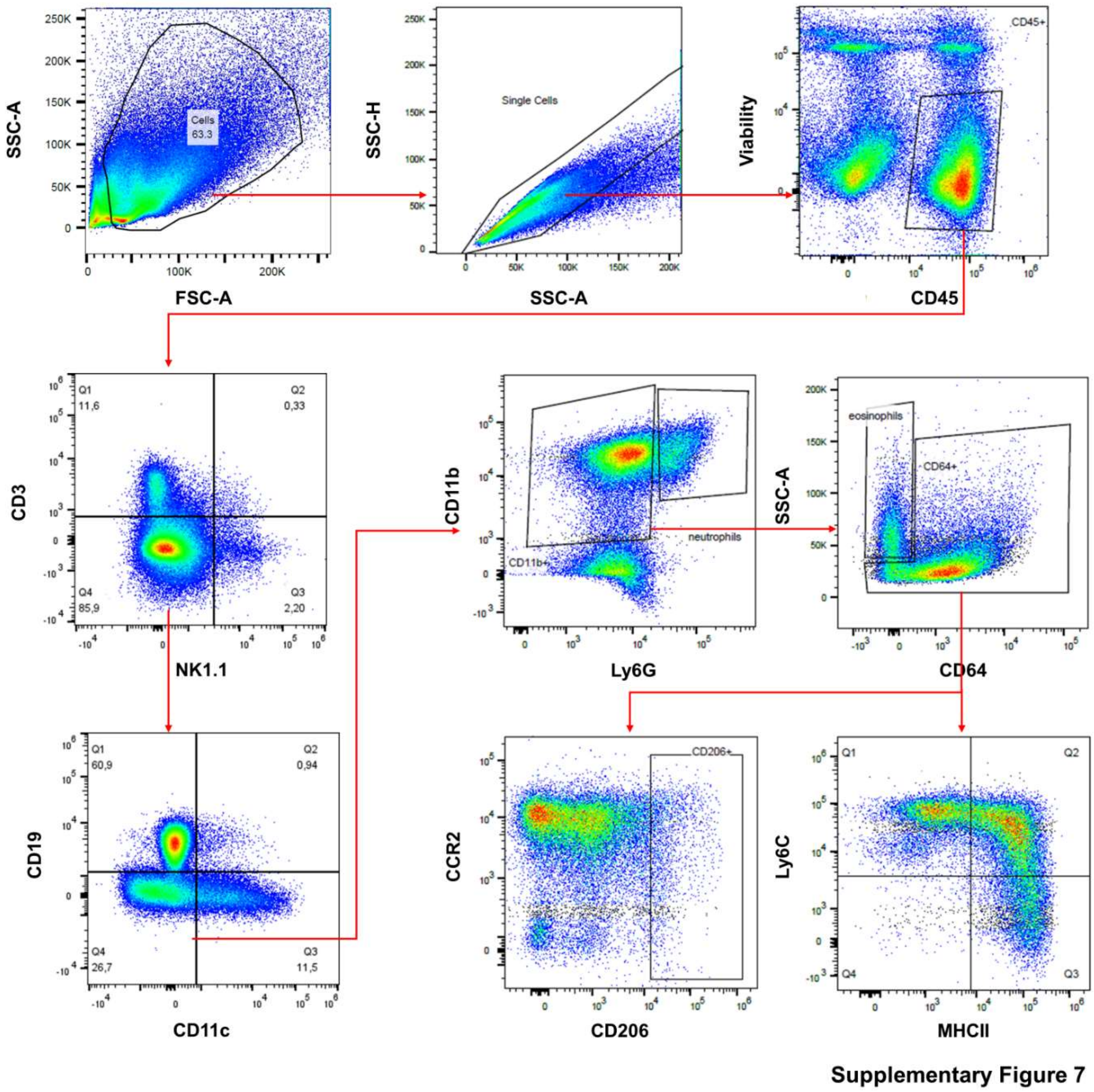

Supplementary Figure 7. Flow-cytometry gating strategy for colonic lamina propria myeloid cells.

Supplementary Table 1.

**Natural Compound**	**Putative TRPM8 affinity (µM)**
Rutin	0.3
**Sesamin**	0.8
Luteolin	1.2
Vitexin	1.6
Kaempferol	1.9
Neochlorogenic acid	2.3
Epigallocatechin	3.8
Rosmarinic acid	4.5
Tricin	5.3
Resveratrol	7.5
Naringenin	8.9
Chlorogenic acid	17.4
**Menthol**	156.1
Rutamarin	3.8
Isochlorogenic acid	7.5
Vitexin compound 1	2.7
P-Cymene	184.7
